# CCR5 structural plasticity shapes HIV-1 phenotypic properties

**DOI:** 10.1371/journal.ppat.1007432

**Published:** 2018-12-06

**Authors:** Philippe Colin, Zhicheng Zhou, Isabelle Staropoli, Javier Garcia-Perez, Romain Gasser, Marie Armani-Tourret, Yann Benureau, Nuria Gonzalez, Jun Jin, Bridgette J. Connell, Stéphanie Raymond, Pierre Delobel, Jacques Izopet, Hugues Lortat-Jacob, Jose Alcami, Fernando Arenzana-Seisdedos, Anne Brelot, Bernard Lagane

**Affiliations:** 1 Viral Pathogenesis Unit, Department of Virology, Institut Pasteur, Paris, France; 2 INSERM Unit U1108, Institut Pasteur, Paris, France; 3 Paris Diderot University, Sorbonne Paris Cité, Cellule Pasteur, Rue du Docteur Roux, Paris, France; 4 AIDS Immunopathogenesis Unit, Instituto de Salud Carlos III, Madrid, Spain; 5 Centre de Physiopathologie Toulouse-Purpan (CPTP), Université de Toulouse, CNRS, Inserm, UPS, Toulouse, France; 6 Grenoble Alpes University, CNRS, CEA, Institut de Biologie Structurale (IBS), Grenoble, France; 7 CHU de Toulouse, Laboratoire de Virologie, Toulouse, France; 8 CHU de Toulouse, Service des Maladies Infectieuses et Tropicales, Toulouse, France; University of North Carolina at Chapel Hill, UNITED STATES

## Abstract

CCR5 plays immune functions and is the coreceptor for R5 HIV-1 strains. It exists in diverse conformations and oligomerization states. We interrogated the significance of the CCR5 structural diversity on HIV-1 infection. We show that envelope glycoproteins (gp120s) from different HIV-1 strains exhibit divergent binding levels to CCR5 on cell lines and primary cells, but not to CD4 or the CD4i monoclonal antibody E51. This owed to differential binding of the gp120s to different CCR5 populations, which exist in varying quantities at the cell surface and are differentially expressed between different cell types. Some, but not all, of these populations are antigenically distinct conformations of the coreceptor. The different binding levels of gp120s also correspond to differences in their capacity to bind CCR5 dimers/oligomers. Mutating the CCR5 dimerization interface changed conformation of the CCR5 homodimers and modulated differentially the binding of distinct gp120s. Env-pseudotyped viruses also use particular CCR5 conformations for entry, which may differ between different viruses and represent a subset of those binding gp120s. In particular, even if gp120s can bind both CCR5 monomers and oligomers, impairment of CCR5 oligomerization improved viral entry, suggesting that HIV-1 prefers monomers for entry. From a functional standpoint, we illustrate that the nature of the CCR5 molecules to which gp120/HIV-1 binds shapes sensitivity to inhibition by CCR5 ligands and cellular tropism. Differences exist in the CCR5 populations between T-cells and macrophages, and this is associated with differential capacity to bind gp120s and to support viral entry. In macrophages, CCR5 structural plasticity is critical for entry of blood-derived R5 isolates, which, in contrast to prototypical M-tropic strains from brain tissues, cannot benefit from enhanced affinity for CD4. Collectively, our results support a role for CCR5 heterogeneity in diversifying the phenotypic properties of HIV-1 isolates and provide new clues for development of CCR5-targeting drugs.

## Author summary

CCR5 regulates host immune responses against pathogens. It also serves as an anchor for R5-tropic strains of HIV-1 to infect immune cells, hence contributing to development of AIDS. CCR5 exists in different forms (*e*.*g*. conformations, oligomerization states), but the mechanisms that govern this diversity and its consequences on the physio-/pathophysio-logical functions of the receptor remain unclear. Because genetically diverse viral isolates populate HIV-1 infected individuals, we asked whether divergent viruses differ in the nature of the CCR5 molecules they use, and if so, whether this accounts for differences in their biological properties. Here we answered in the positive to both questions. We also identified CCR5 oligomerization as a key process regulating the receptor conformational diversity, the extent to which HIV-1 envelope glycoproteins bind to target cells and viral entry efficacy. From a functional standpoint, the nature/quantity of the receptor populations that are used by HIV-1 isolates regulates the type of cells they can infect and their ability to escape inhibition by CCR5 ligands. This study thus represents a step forward toward understanding of the mechanisms that regulate CCR5 diversity and its implications on the virus biological properties while opening new avenues for the development of drugs targeting CCR5.

## Introduction

CC Chemokine Receptor 5 (CCR5) is a G Protein-Coupled Receptor (GPCR) that regulates immune functions [1–3]. Members of the GPCR family exist in structurally diverse forms resulting from differential post-translational modifications and oscillation between different conformational and oligomerization states [4–8]. CCR5 itself oscillates between inactive and active conformations [9, 10]. The existence of multiple CCR5 populations has been inferred from the observation that structurally distinct CCR5 ligands recognize different proportions of the receptor at the cell surface [11–15]. The nature and/or quantity of the CCR5 conformations may vary between different cell types [11, 14]. CCR5 conformation is regulated by several factors, including signaling effectors and membrane lipid composition [12, 13, 16, 17], the receptor homo- and hetero-oligomerization [18–20] and the binding of ligands [21–26]. Computational analysis predicted that CCR5 can adopt an ensemble of twenty low-energy conformations, each of which being differentially favored by distinct ligands and receptor mutations [27]. However, the nature and the functional properties of the different CCR5 populations remain elusive.

CCR5 is also hijacked by R5-tropic strains of HIV-1 for entry into immune cells [28–30]. HIV-1 entry begins with the interaction of the surface subunit (gp120) of the viral envelope glycoprotein (Env) with cellular CD4. Regions in gp120 (the so-called bridging sheet and the V3 loop) are then created/exposed and interact with distinct domains of CCR5 used as coreceptor, including the N-tail and the second extracellular loop (ECL2). This triggers fusion between the viral and cell membranes [31–36]. HIV-1 Env is subjected to high intra- and inter-patient structural diversity [37–39]. Sequence variability in Env allows HIV-1 to escape host immune responses and develop resistance to entry inhibitors. Distinct viral isolates may also differ in the way they utilize CCR5 [40]. However, it is not demonstrated whether different HIV-1 strains use differentially the diverse CCR5 populations and if so, whether a link exists between the nature of the CCR5 forms used by a virus and its role in HIV infection. Actually, the capacity of CCR5 ligands to inhibit HIV-1 entry may not correlate with the extent to which they bind to CCR5 [11], hence indirectly suggesting that HIV-1 itself uses only subsets of cell surface receptors. Here we show that distinct HIV-1 strains may differ in the nature of the CCR5 molecules to which they bind, and this is likely to steer some of their phenotypic properties such as cellular tropism and capacity to escape inhibition by CCR5 ligands. This study thus highlights the relationship between CCR5 structural plasticity and diversification of HIV-1 phenotypic properties and has implications on the development of HIV-1 entry inhibitors targeting CCR5.

## Results

### Distinct HIV-1 gp120s recognize different quantities of cell surface CCR5

To investigate whether different HIV-1 strains bind differentially to distinct CCR5 subpopulations, we first measured binding of ^35^S-labeled gp120s to CCR5-expressing HEK 293T cells (HEK-R5 cells) in the presence of soluble CD4 (sCD4), as previously detailed [41]. Most of these gp120s were from biological virus clones isolated from peripheral blood mononuclear cells (PBMCs) of four treatment-naïve, HIV-positive individuals longitudinally followed-up. From each individual, PBMCs were collected at two time points: during the chronic stage of infection (Envs #1, 25, 38, and 50) and around the time of AIDS diagnosis (Envs #10, 34, 48, 58, and 59). These Envs were confirmed to be R5-tropic by phenotypic tropism assays. The other R5 Envs used here are Bx08 and 1f (S4 Fig) [9, 42], and the macrophage- (M-) and T-cell (T-) tropic Envs JR-FL and JR-CSF [43].

**Fig 1:**
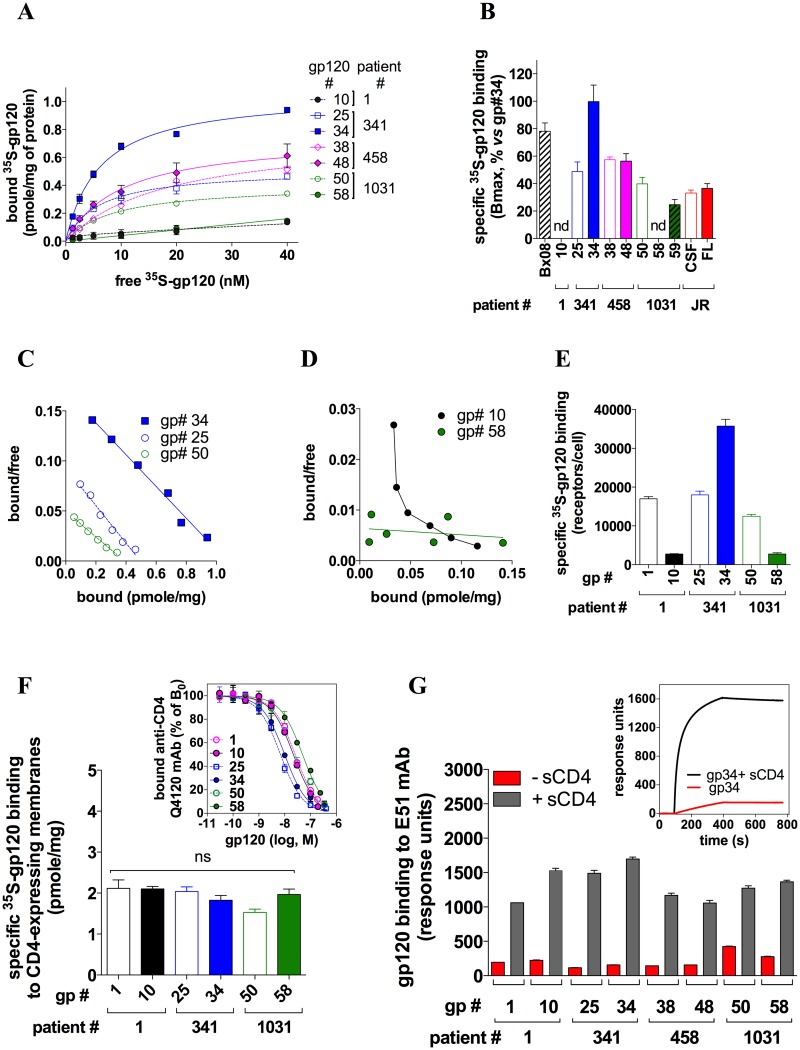
Distinct gp120s show discordant binding levels to CCR5 but not to CD4 or the E51 mAb. **A** Saturation binding curves of ^35^S-gp120s to HEK-R5 membranes in the presence of 400 nM sCD4. Specific binding of gp120s was deduced by subtracting from total binding the non-specific binding (NSB). NSB was measured using 10 μM of the CCR5 antagonist maraviroc (MVC) and was observed to increase linearly with the concentration of ^35^S-gp120, as expected in the case of low affinity binding. Similar results were obtained when measuring NSB on parental HEK 293T cells. Relative to total binding, mean NSB values at a concentration of 10 nM gp120 represented 59.3%, 27.3%, 12%, 24.3%, 20.1%, 17% and 54.3% for gp120 #10, 25, 34, 38, 48, 50 and 58, respectively. These differences proved mainly due to differences in the extents of specific binding, and not to differences in NSB between the distinct gp120s. One representative experiment out of at least three independent experiments performed in duplicate is shown. On the whole, three to four independent batches of purified gp120s were used for these experiments, with similar results. Data points were described by a one-site (gp120 #25, 34, 38, 48, 50) or a two-site binding model (gp120 #10) or linear regression (gp120 #58). **B** The B_max_ values (means ± SD) for the gp120s were deduced from the saturation curves using the Prism Software. n.d.: not determined. The binding levels of JR-FL gp120 and gp120 #34 to CCR5 were compared using both proteins at their K_D_ (*i*.*e*. 4.35 nM for JR-FL gp120 [45] and 7 nM for gp120 #34 (S1 Table)). Binding of JR-CSF gp120 was also determined at 4.35 nM, although the K_D_ value for this protein is not known. **C, D** The Scatchard transformations of the binding data were deduced using Prism. For the sake of clarity, the linear Scatchard plots for gp120 Bx08, #38, #48 and #59 are not represented. **E** Specific binding of 10 nM ^35^S-gp120 to intact HEK-R5 cells (1.10^6^), with 200 nM sCD4 (n = 2). Relative to total binding, mean NSB values (determined as in A) represented 19%, 27.9%, 6.9%, 3.4%, 10% and 39.8% for gp120 #1, 10, 25, 34, 50 and 58, respectively. **F** Specific binding to HEK-CD4 cell membranes of ^35^S-gp120s used at a concentration equal to their K_I_ for CD4 (means ± SEM of 3 experiments). K_I_ were deduced from competition binding experiments of the anti-CD4 Q4120 mAb by unlabeled gp120 (inset). NSB was determined using parental HEK cells and represented less than 10% of total binding. ns: P > 0.05 using unpaired two-tailed Student *t* test. **G** SPR measurement of the binding of 100 nM gp120 to the mAb E51 immobilized on a CM4 sensorchip, alone or with 200 nM sCD4. Results are means ± SEM (n = 2). Inset: Representative sensorgrams for gp120 #34.

HEK-R5 cells express high enough amounts of CCR5 (200,000 receptors/cell, [9]) so that binding of HIV-1 gp120s could be determined with a high ratio between specific binding (*i*.*e*. binding to CCR5) and non-specific binding (legend of Fig 1). They also exhibit heterogeneous conformations of CCR5 at their surface [9, 12]. Saturation binding experiments of ^35^S-gp120s to HEK-R5 cells revealed that the maximum number of binding sites (B_max_) varied from one gp120 to another, indicating that the glycoproteins do not recognize the same amounts of the CCR5 molecules (Fig 1A, 1B and 1E and S1 Table). JR-FL and JR-CSF gp120s, although exhibiting different cellular tropisms, showed similar binding levels (Fig 1B). Scatchard transformation of binding data was linear for most of the gp120s (Fig 1C), suggesting that they bind to a homogeneous population of receptors. Equilibrium dissociation constants K_D_ of most of the gp120/CCR5 interactions are in the range of few to some tenths of nM (S1 Table). These values are similar to those of the lab-adapted gp120s BaL, JR-FL and YU2 [44–46]. Two exceptions are gp120 #58 that displays non-saturable, low-affinity binding to CCR5 and gp120 #10 whose Scatchard plot is non-linear, suggesting that the glycoprotein binds with different affinities to multiple CCR5 populations (Fig 1D).

We next investigated whether the gp120s show divergent binding levels to CD4. Competition binding assays of the anti-CD4 mAb Q4120 to CD4-expressing HEK cells indicated that the gp120s have similar nM affinity constants K_I_ for the receptor (Fig 1F, **inset** and S1 Table). These affinity constant values are comparable to those of other R5 gp120s (YU2, BaL, JR-FL and JR-CSF) reported by us [41] and others [47–50]. Then, we measured the binding levels of the ^35^S-gp120s to HEK-CD4 cells (S1 Fig) or membranes from these cells (Fig 1F) at a concentration of the gp120s equal to their K_I_, *i*.*e*. a concentration at which half of the CD4 molecules is occupied. In contrast to CCR5, the gp120s had comparable levels of binding to CD4. Next we investigated if the different capacities of the gp120s to bind to CCR5 could be due to the fact that they are not properly folded when bound to CD4. By surface plasmon resonance experiments, we measured the binding of the gp120s to the mAb E51 (Fig 1G), which targets regions in gp120 that are induced by CD4 and overlap the CCR5 binding site [42]. The gp120s bound efficiently to the mAb in the presence, but not in the absence, of sCD4. This indicates that sCD4 has triggered conformational changes in the gp120s exposing the CCR5 binding sites. Overall, these results indicate that the divergent binding levels of gp120s to CCR5 do not result from impaired binding to CD4 or incorrect structural modifications induced upon CD4 binding.

### The number of CCR5 molecules that are recognized by different HIV-1 gp120s varies differentially between different cell types

We speculated that the different gp120s could bind differentially to distinct CCR5 populations that exist in varying amounts at the cell surface. To address this issue, we first investigated whether the extents to which the different gp120s bind to CCR5 are cell-type dependent. Indeed, the nature and the relative proportions of the CCR5 populations may vary between cell types [11, 14]. We thus measured the binding levels of ^35^S-gp120s to CCR5-expressing U87 cells (S2A Fig). Some gp120s labeled equal amounts of the receptors in HEK-R5 and U87-R5 cells (gp120 #1, 25 and 38) while others showed either increased (gp120 #34 and 50) or decreased (gp120 #10, 48 and 58) binding to U87-R5 cells, compared to HEK-R5 cells. We next compared the binding of ^35^S-gp120 #25 and #34 to CCR5 on intact (S2C Fig) or membranes from (S2B Fig) activated CD4^+^ T-lymphocytes and monocyte-derived macrophages (MDMs). The differences in the levels of binding between gp120 #25 and 34 were less marked with T-cells than with MDMs. Altogether, these data indicate that the different gp120s differentially target distinct CCR5 populations whose relative proportions vary between cell types.

### Interaction of HIV-1 gp120s with antigenically distinct conformations of CCR5

We next studied if the differences in the binding levels between the different gp120s are related to differential recognition of antigenically distinct conformations of CCR5 [11, 13–15]. We thus performed competition binding assays of the ^35^S-gp120s to HEK-R5 or U87-R5 cells by the anti-CCR5 mAbs CTC5, 2D7 or 45531. These mAbs react with distinct motifs and conformations of CCR5 (S3A Fig and refs [11, 14]). For the sake of clarity, detailed analysis of the results is presented in the accompanying supplemental S1 Text. Briefly, the main conclusions that have emerged from the experiments in S3B, S3C and S3D Fig are the following. First, we observed that different gp120s may be differentially sensitive to inhibition by some of the mAbs. We interpreted this as strong indication that the gp120s bind differentially to distinct CCR5 conformations, which differ in their ability to be recognized by the mAbs. We also found that some gp120s are only partially inhibited by some mAbs, suggesting that these gp120s recognize at least two distinct CCR5 populations, one interacting with these mAbs, the other that does not. Alternative explanation can however be considered (S1 Text). Finally, sensitivity of the gp120s to the mAbs varies between HEK-R5 (S3C Fig) and U87-R5 (S3D Fig) cells, suggesting that the gp120s do not bind the same CCR5 conformations in both cell types.

Altogether, these results show that the gp120s may target differentially distinct antigenic populations of CCR5, and this can contribute to their divergent binding levels to CCR5-expressing cells (Fig 1). In S3 Fig however, it is also apparent (in particular with U87-R5 cells) that some gp120s can share similar sensitivities to anti-CCR5 mAbs while showing divergent binding levels to CCR5 (*e*.*g*. compare S2A and S3D Figs and see S1 Text). This indicates that distinct CCR5 populations can vary in the nature of the gp120s to which they bind while sharing similar antigenic properties.

### HIV-1 uses a fraction of the CCR5 forms for entry into cells

Next, we investigated whether the distinct CCR5 forms that bind gp120s are also used by viruses for entry into cells. NL4-3-derived virus clones pseudotyped with some of the different full-length (gp160) Envs used above, or gp160 from the JR-FL or JR-CSF strains, were investigated for their sensitivity to inhibition by the mAbs 2D7, 45531 or CTC5 in single-round infection assays of U87-CD4-CCR5 cells (S3E Fig). MAb 2D7 showed the most effective antiviral activity among the tested mAbs (≥ 75% inhibition for all viruses). This contrasts with the fact that it inhibits more weakly the binding of gp120s to U87-R5 cells, as compared to 45531 and CTC5 (S3D Fig). In the same line, 45531 and CTC5 efficiently prevented the different Envs from binding to U87-R5 cells (S3D Fig), but they only slightly inhibited infection by some of the pseudotyped viruses (viruses #1, #34, #58 and JR-FL).

Thus, each of the three mAbs may differ in its ability to inhibit gp120 binding and HIV-1 viral entry. One hypothesis that explains this result is that only a subset of the CCR5 forms to which gp120s bind are used for viral entry. This is in agreement with previous results suggesting that HIV-1 uses only some of the antigenic forms of CCR5 for entry into cells [11]. In addition, we found here that the CCR5 forms that are used by the different viruses are heterogeneous and can be discriminated on the basis of their differential reactivity to mAbs 45531 and CTC5, but not to 2D7. The binding experiments with gp120s suggest, however, that differences in sensitivity of viruses to mAbs may be only partly reflective of heterogeneity of the CCR5 forms used for viral entry.

### Distinct primary HIV-1 envelope glycoproteins differ in the way they engage CCR5

**Fig 2:**
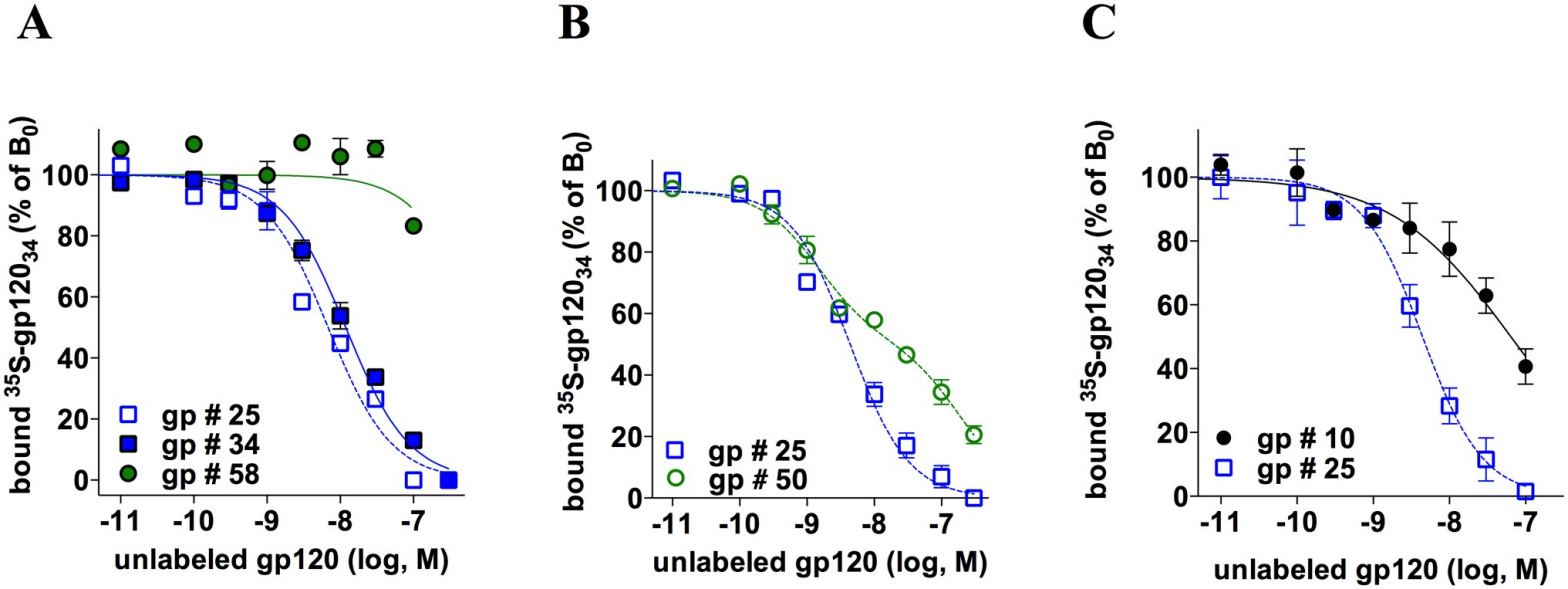
Different HIV-1 gp120s show different modes of binding to CCR5. The panels show competition binding experiments of 10 nM ^35^S-gp120 #34 to HEK-R5 membranes by increasing concentrations of unlabeled gp120 #34 (**A**), #58 (**A**), #50 (**B**) and #10 (**C**). In all experiments, unlabeled gp120 #25 was used as an internal control (**A**, **B** and **C**). Representative experiments out of 3 independent experiments performed in duplicate are shown. Results (means ± SEM) were normalized for non-specific binding (0%) and specific binding in the absence of competitor (100%, B_0_). In the panels, data points are fitted according to a one-site (gp120 #25, 34 and 58) or a two-site (gp120 #50, *F* value = 43.9; *p* < 0.0001) competitive binding model or a sigmoidal dose-response model with a variable slope (gp120 #10). The Hill slope (n_H_) values were deduced from fitting of the competition curves according to a sigmoidal dose-response model with a variable slope.

To confirm that different gp120s bind to/stabilize distinct CCR5 subsets, we measured the binding of ^35^S-gp120 #34 to HEK-R5 membranes in the presence of unlabeled gp120 #10, #25, #34, #50 or #58 (Fig 2A, 2B, 2C and S1 Table). As detailed in S2 Text, results indicated that gp120 #10 and gp120 #50 bind with distinct affinities to multiple ^35^S-gp120 #34-binding receptors. In contrast, gp120 #58 failed to displace the binding of ^35^S-gp120 #34, indicating that gp120 #58 and ^35^S-gp120 #34 do not bind to the same CCR5 populations. We also observed that gp120 #25 and gp120 #34 were equally potent and efficient in displacing the binding of ^35^S-gp120 #34 (Fig 2A). This result was unanticipated because gp120 #25 binds less receptors than gp120 #34 in the saturation experiments (Fig 1). Similar results with other gp120s are shown in S4 Fig. As discussed in S2 Text, this suggests that both gp120s bind to the same receptor populations but with divergent stoichiometries. In the context where CCR5 exists as dimers/oligomers [20, 51], a model recapitulating the above results considers that HIV-1 gp120 could form both 1:2 and 2:2 stoichiometric complexes with CCR5 dimers, the frequency of each of these two situations depending on the nature of the gp120. In the first situation (1:2 stoichiometry), a single gp120 would interact concomitantly with both protomers of the CCR5 dimer, each of them providing different interaction sites for the gp120 (*e*.*g*. the N-tail could originate from protomer 1 and ECL2 from protomer 2), hence forming the whole binding site for the glycoprotein. In the second situation (2:2 stoichiometry), the two protomers would provide two identical and independent binding sites per dimer for the gp120. We propose that gp120 #25 favors the 1:2 stoichiometry at the expense of the 2:2 stoichiometry, and conversely for gp120 #34. This would explain how gp120 #25 could inhibit the binding of gp120 #34 by a competitive mechanism while showing a lower B_max_ value in saturation binding assays. This model also considers oligomeric CCR5 as a particular CCR5 population that could play a major role in diversification of the modes of gp120/CCR5 interactions (NB: For simplicity, oligomeric CCR5 will be referred to as “dimer” or “oligomer” in the rest of the paper).

### Dimerization of CCR5 differentially modulates CCR5 recognition by distinct gp120s

**Fig 3:**
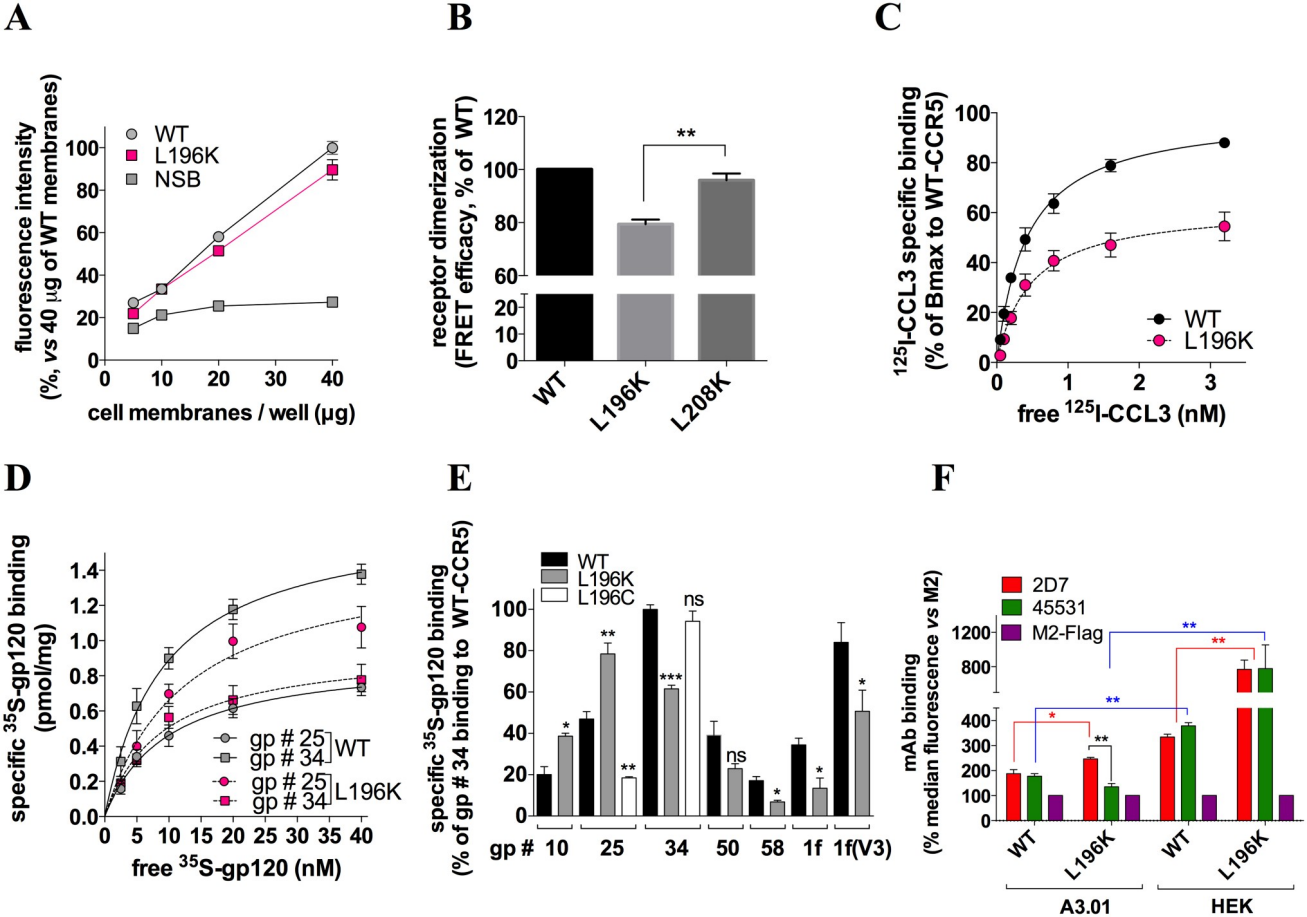
Distinct HIV-1 gp120s are differentially sensitive to CCR5 dimerization. **A** Membranes from HEK 293 cells transfected with FLAG/SNAP-tagged WT-CCR5 or L196K-CCR5 cDNA show similar receptor expression levels. Fluorescence intensity (at 620 nm) of the SNAP substrate BG-Lumi4-Tb bound to receptors increases linearly with membrane quantity, in contrast to non specific binding (NSB) to parental membranes. **B** FRET efficacy was measured between SNAP- and CLIP-tagged receptors labeled with lumi4-Tb and d2, respectively, and expressed as the ratio of the fluorescence intensities at 665 nm (d2) and 620 nm (Lumi4-Tb) (λ_ex_ = 320 nm). HEK cells were transfected in such a way that WT-CCR5, L196K-CCR5 and L208K-CCR5 have similar cell surface expression levels and that the FRET acceptor(CLIP)/donor(SNAP) ratio is non-saturating and kept constant in all experiments. Mutation of Leu-196 reduces CCR5 dimerization, in contrast to Leu-208 that resides outside the dimerization interface [52] (n = 3). **C**, Saturation binding curves of the CCR5 chemokine ^125^I-CCL3 to FLAG/SNAP-tagged WT-CCR5- or L196K-CCR5-expressing membranes. Results indicate that L196K-CCR5 has a 36%-fold lower Bmax value than WT-CCR5 (2.5 *vs* 3.9 pmole/mg of protein, respectively). In the range of the ^125^I-CCL3 concentrations tested, however, similar affinity constant values were deduced from the curves for WT- and L196K-CCR5 (K_D_ = 0.36±0.18 and 0.47±0.13 nM, respectively). **D** Saturation binding curves of ^35^S-gp120 #25 or #34 to FLAG/SNAP-tagged WT-CCR5- or L196K-CCR5-expressing membranes. **E** Specific binding of 10nM ^35^S-gp120/sCD4 to WT-CCR5-, L196K-CCR5- or L196C-CCR5-expressing membranes. * *P*<0.05; ** *P* <0.01; *** *P* <0.001, *vs* binding to WT-CCR5 in unpaired two-tailed Student *t* test. Results (means ± SEM, n = 3–6) are expressed as % specific binding relative to ^35^S-gp120 #34 binding to WT-CCR5. **F** Binding of mAbs 2D7, 45531 or anti-Flag M2 to FLAG/SNAP-tagged WT- or L196K-CCR5-expressing A3.01 or HEK cells was revealed by AlexaFluor 647-conjugated secondary Ab and flow cytometry analysis (n = 3).

To investigate the role of CCR5 di-/oligo-merization in gp120 binding, we compared the ligand binding properties of FLAG/SNAP-tagged wild-type (WT) and L196K CCR5. Indeed, we recently identified that Leu-196 is part of the CCR5 dimerization interface [52]. The L196K mutation results in receptor dimers that are less stable and involve alternative dimerization interfaces, as compared to WT CCR5 ([52] and Fig 3B). Receptors were expressed in HEK 293 cells and membranes containing similar amounts of either WT- or L196K-CCR5 (Fig 3A) were prepared. In this context, L196K-CCR5 had a lower capacity to bind the chemokine ^125^I-CCL3 than WT-CCR5 (Fig 3C, see the legend for details). This suggests that CCR5 dimerization plays a role in high-affinity binding of agonist chemokines. Alternatively, the mutation might alter the equilibrium between high- and low-affinity conformations of the receptor, regardless of its dimerization status.

The gp120 binding levels varied differentially between WT- and L196K-CCR5. In saturation binding assays to WT-CCR5 (Fig 3D), ^35^S-gp120 #25 labeled fewer receptors than ^35^S-gp120 #34. Strikingly, we observed the reverse situation with L196K-CCR5. As compared to WT-CCR5, we found a rise in the B_max_ value of ^35^S-gp120 #25 to L196K-CCR5, while that of ^35^S-gp120 #34 was dramatically decreased. Other gp120s behaved similarly to gp120 #34 or gp120 #25, albeit to varying degrees, in that they bind less or more efficiently L196K-CCR5, respectively, than WT-CCR5 (Fig 3E).

**Fig 4:**
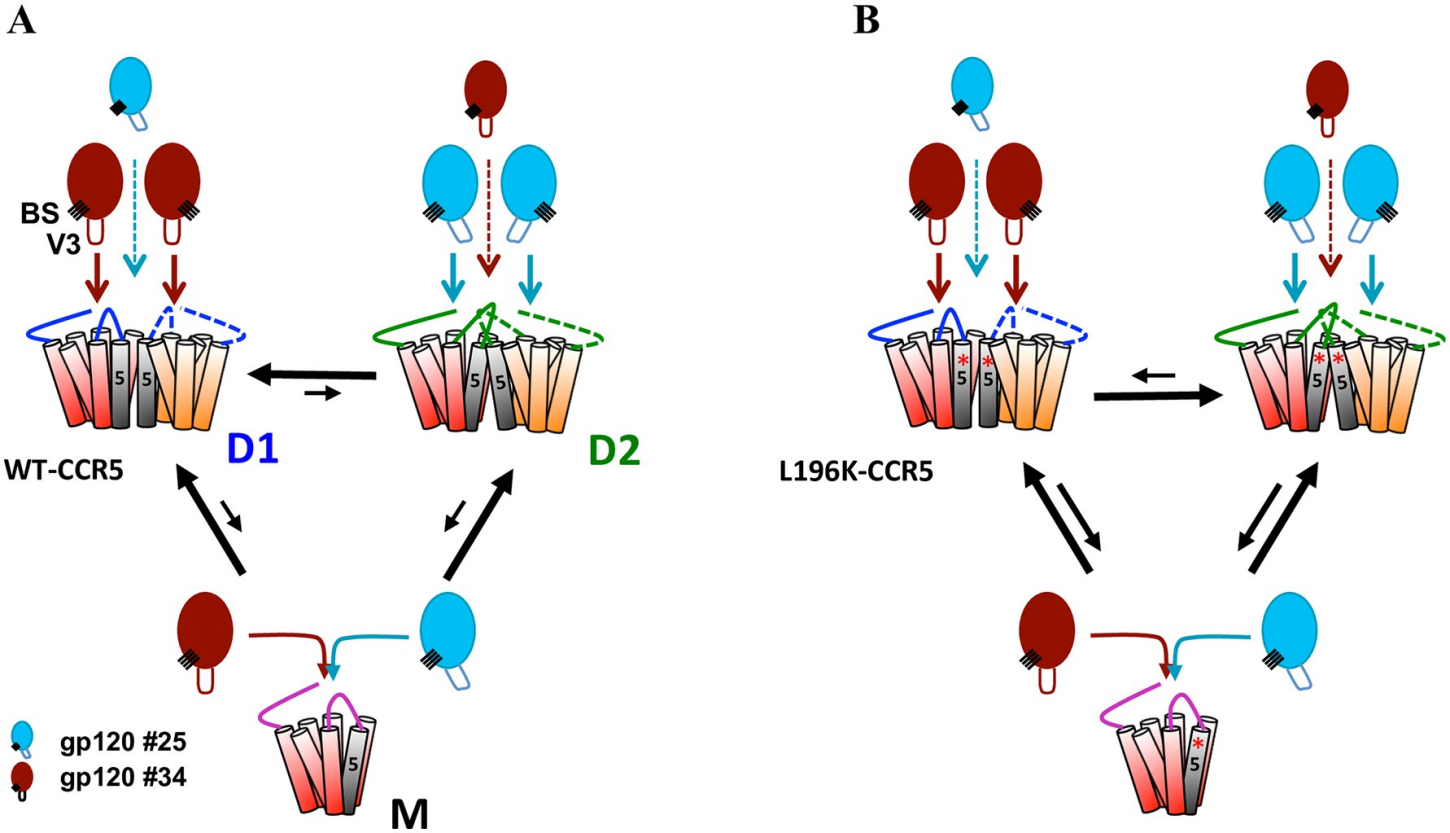
Model for the role of CCR5 dimerization in gp120 binding. CCR5 exists in at least two distinct dimer conformations (D1 and D2), which coexist with receptor monomers (M). The orientations of TM5 and ECL2 are different in D1 and D2. Depending on the nature of the tip of the V3 loop (V3) and perhaps its distance/position relative to the bridging sheet (BS), distinct gp120s differ in the way they engage D1 and D2 while binding similarly receptor monomers. Some gp120s (*e*.*g*. gp120 #34, brown colored) bind D1 and D2 in 2:2 and 1:2 stoichiometries, respectively, and conversely for other gp120s (*e*.*g*. gp120 #25, blue colored). **A** In WT-CCR5-expressing cells, D1 is predominant over D2 and monomers explaining why the number of gp120 #34 molecules bound to these cells is higher than that of gp120 #25 molecules (Figs 1 and 3 and S2 Fig). **B** L196K-CCR5 preferentially adopts the D2 conformation, in which the conformation of ECL2 differs, compared to that in D1. D2 binds gp120 #25 and #34 in 2:2 and 1:2 stoichiometries, respectively. This explains why the number of gp120 #25 molecules bound to L196K-CCR5-expressing cells is higher than that to WT-CCR5-expresing cells, and conversely for gp120 #34 (Fig 3D and 3E). Equilibrium arrows in **A** and **B** show that the proportion of dimers is decreased in the case of L196K-CCR5, as compared to WT-CCR5.

The model in Fig 4 recapitulates these results. It posits that CCR5 dimers (D) and monomers (M) coexist, in agreement with current knowledge on GPCR dimerization [6, 53], and that the proportion of dimers is reduced in L196K-CCR5-expressing cells (in line with Fig 3B and ref. [52]). Gp120 binding to WT- and L196K-CCR5 gave similar K_D_ (S1 Table) and proceeded according to a one-site binding model (Fig 3D), indicating that the gp120s bind both receptors with similar affinities, regardless of their oligomerization state. In this context, considering that gp120 #25 and #34 target the same CCR5 molecules (Fig 2A), the results in Fig 3D suggest that both gp120s have different interaction stoichiometries with receptor dimers that vary differentially between WT- and L196K-CCR5. While results are consistent with gp120 #25 favoring the 1:2 at the expense of the 2:2 stoichiometry with WT-CCR5 dimers (Fig 4A), the reverse situation seems to take place with L196K-CCR5 dimers (Fig 4B), and conversely for gp120 #34 (Fig 4, see the legend for details). The results in Fig 3E suggest that similarly the other gp120s may establish different stoichiometries with WT- and L196K-CCR5. These results thus suggest that changes in the gp120/dimer interaction stoichiometries contribute to the apparent differences in the binding capacities of R5 gp120s to CCR5 (Fig 1).

Further experiments suggested that WT- and L196K-CCR5 differ in how they bind the gp120s because of different ECL2 conformations. Indeed, both receptors bind differentially the anti-ECL2 mAbs 2D7 and 45531, in a cell type-dependent manner (Fig 3F). In the same line, the binding levels of gp120s to CCR5 depend on the composition of the tip of the gp120 V3 loop, *i*.*e*. the main determinant for interaction with ECL2 (Fig 3E). This suggests that gp120s form different stoichiometric complexes with CCR5 dimers owing to differences in the conformation of their V3 loop.

We speculated that favoring CCR5 dimerization might decrease the binding level of gp120s more efficiently when they bind dimers in a 1:2 stoichiometry. We thus performed binding experiments of ^35^S-gp120s to the L196C-CCR5 mutant, which has an increased propensity to form symmetrical dimers and higher-order oligomers [52]. The extent to which ^35^S-gp120 #25 binds L196C-CCR5 was dramatically reduced, as compared to WT-CCR5 (Fig 3E). In contrast, the L196C mutation poorly changed the binding of ^35^S-gp120 #34, reinforcing the notion that ^35^S-gp120 #25 and #34 bind CCR5 dimers preferentially in 1:2 and 2:2 stoichiometries, respectively.

### Impairment of CCR5 dimerization improves HIV-1 entry into T-lymphocytes

**Fig 5:**
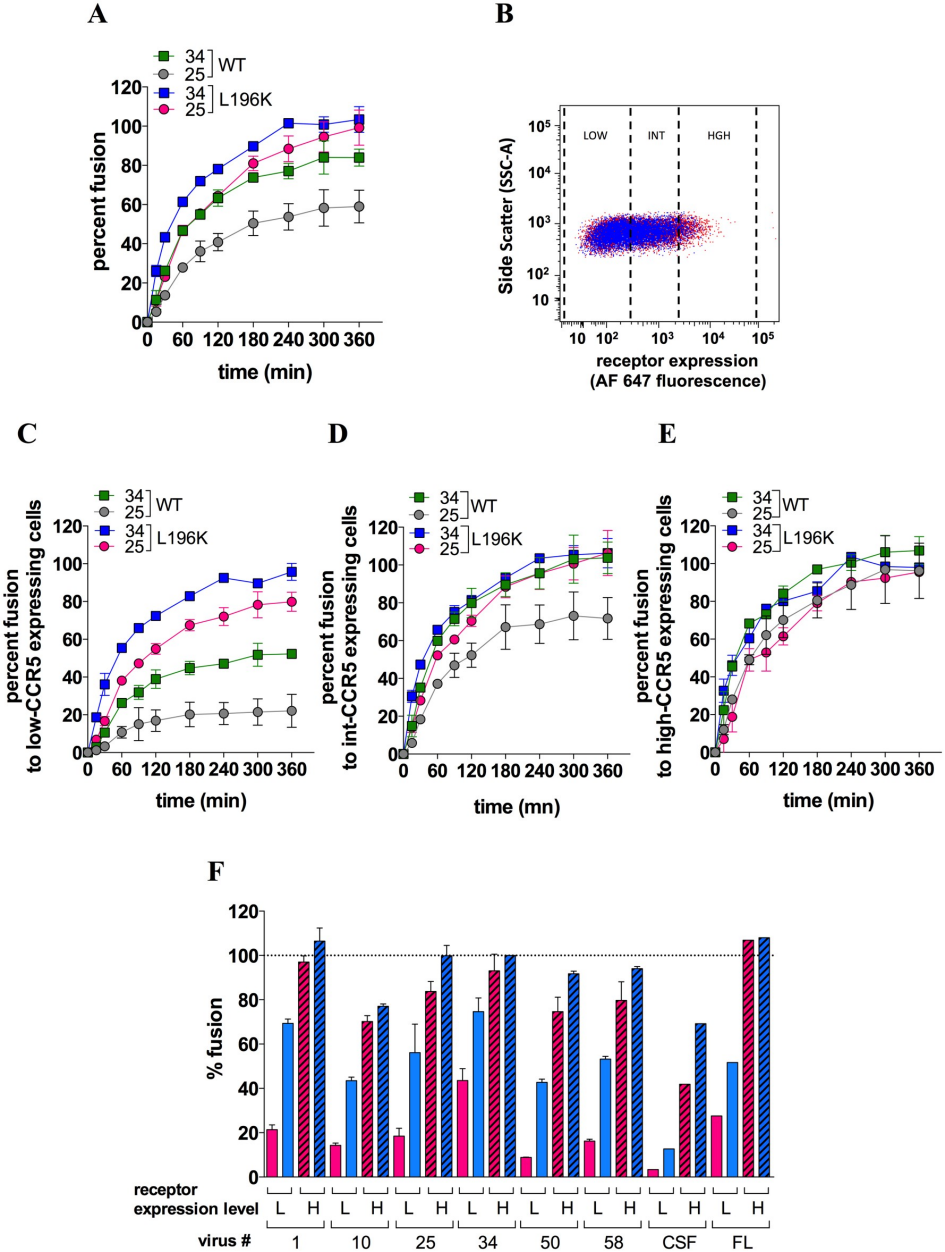
Role of CCR5 dimerization in HIV-1 entry into T-cells. **A** Fusion kinetics of BlaM-vpr-containing virus #25 or 34 with CD4+ A3.01 T-cells expressing FLAG/SNAP-tagged WT-CCR5 or L196K-CCR5. Data points represent means ± SEM of 2 independent determinations (out of 5). **B** Gating strategy of A3.01 cells expressing WT-CCR5 (red) or L196K-CCR5 (blue) at a low (GMFI = 111 and 123 for WT-CCR5 and L196K-CCR5, respectively), intermediate (599 and 533) or high level (2903 and 2382). Shown are representative flow cytometry plots of cell side scatter *vs* receptor expression level revealed with the AlexaFluor 647-conjugated anti-FLAG mAb M2. On the whole cell populations, both receptors were expressed at similar expression levels (GMFI = 362 and 312 for WT-CCR5 and L196K-CCR5, respectively). Comparable results were obtained using the unconjugated M2 revealed by an AlexaFluor 647-conjugated goat anti-mouse IgG (GAM) (GMFI = 5871 and 3342) **C**, **D** and **E** The virus-cell fusion experiments shown in **A** were analyzed with A3.01 T-cells expressing low (**C**), intermediate (**D**) or high (**E**) cell surface level of either WT- or L196K-CCR5. **F** Levels of fusion at 3 h for the indicated viruses were measured with A3.01 T-cells expressing low (L) or high (H) level of either WT- (red bars) or L196K-CCR5 (blue bars). Results are means ± SEM of 3 independent determinations (except for the non-M and M-tropic viruses JR-CSF and JR-FL where n = 1). In panels **A**, **C**, **D**, **E** and **F**, results are expressed as percents of fusion relative to the maximum extent of fusion (F_max_) of virus #34 with L196K-CCR5-expressing cells. This F_max_ value, expressed as the percentage of cells containing BlaM-vpr, ranged between 40–60% and did not vary with the receptor expression level.

We next investigated whether the differences in the way dimerization influences gp120 binding to CCR5 translate into differences in efficiency of viral entry. Using the BlaM-vpr/CCF2 virus-cell fusion assay [42], we measured the ability of NL4-3-derived virus clones pseudotyped with gp160 #25 (virus #25) or #34 (virus #34) to fuse with A3.01 T-cells (CD4+/CCR5-) expressing comparable levels of WT- or L196K-CCR5 (Fig 5B). Fusion to WT-CCR5-expressing cells occurred at the same rate for both viruses (S5 Fig) and reached a plateau value (F_max_) that is ≈ 30 percent-fold lower for virus #25 (grey colored curve) compared to virus #34 (green colored curve) (Fig 5A).

We then speculated that increasing CCR5 expression might rescue the fusion capacity of virus #25. To address this issue, we coupled the fusion assay with immunolabeling of CCR5 and then analyzed fusion to cell populations expressing low, intermediate or high levels of CCR5 (Fig 5B, 5C, 5D and 5E). F_max_ for virus #25 was minimal with low-CCR5-expressing cells, representing less than 40% of that of virus #34 (Fig 5C), and then gradually increased with the CCR5 expression level up to values approximating those of virus #34 (Fig 5D and 5E). F_max_ for virus #34 also increased with the CCR5 expression level but less deeply, as compared to virus #25. Overall, these data indicate that fusion of virus #25 is more dependent on the CCR5 expression level than fusion of virus #34, in agreement with fewer Env #25 molecules binding T-cells as compared to Env #34 (S2B and S2C Fig).

Fusion of both viruses to L196K-CCR5-expressing A3.01 T-cells proceeded at a similar rate compared to WT-CCR5-expressing cells (S5 Fig), consistent with gp120 #25 and #34 binding WT-CCR5 and L196K-CCR5 with similar affinities (Fig 3D). L196K-CCR5 rendered fusion of viruses #25 and #34 less dependent upon the receptor expression level. Indeed, fusion of virus #34 was maximal regardless of the receptor expression level (Fig 5C, 5D and 5E). Virus #25 behaved similarly as virus #34, except with low-CCR5-expressing cells where fusion was slightly (15%-fold) decreased.

The fact that virus #34 enters more efficiently into L196K-CCR5-expressing cells was however unexpected because gp120 #34 binds less efficiently L196K-CCR5 dimers than WT-CCR5 dimers (Figs 3 and 4). Other R5 viruses displayed improved fusion to L196K-CCR5-expressing cells regardless of how they interact with the receptor dimers (Fig 5F), suggesting that the receptor dimers do not contribute much to HIV-1 entry into T-cells. A similar trend was observed using HEK-CD4 cells as target cells (S6 Fig). Overall, these data suggest that HIV-1 uses predominantly CCR5 monomers to fuse to T-cells and that fusion to L196K-CCR5-expressing cells is improved due to decrease in the proportion of dimers, as compared to WT-CCR5 expressing cells.

### CCR5 plasticity also influences HIV-1 entry into macrophages

Macrophages play a key role in HIV-1 infection pathogenesis [54–57]. Yet, many R5-viruses are inefficient at infecting these cells. Highly macrophage- (M-)tropic HIV-1 strains are predominant in brain tissue [58–60], where they have evolved to use the low CD4 densities that feature the local target cells (macrophages and microglial cells) [61]. These viruses exhibit high efficiency of CD4 use, as revealed by increased sensitivity to inhibition by sCD4 [43, 49, 59, 62] and the capacity to infect Affinofile cells expressing minimum density of CD4 (CD4^low^ Affinofile cells) [59, 60, 63]. Infection of brain long-lived cells in some patients can shelter the virus for a long period of time, which is reflected by slow decay of viral load in the cerebrospinal fluid (CSF) under cART [61]. Such highly M-tropic viruses are rather rare in the blood and immune tissues, where R5 isolates are adapted to infect T-cells on which CD4 density is higher [63]. R5 T-tropic isolates showing a certain degree of infection of macrophages have nevertheless been described in certain occasions [58, 60, 63, 64].

**Fig 6:**
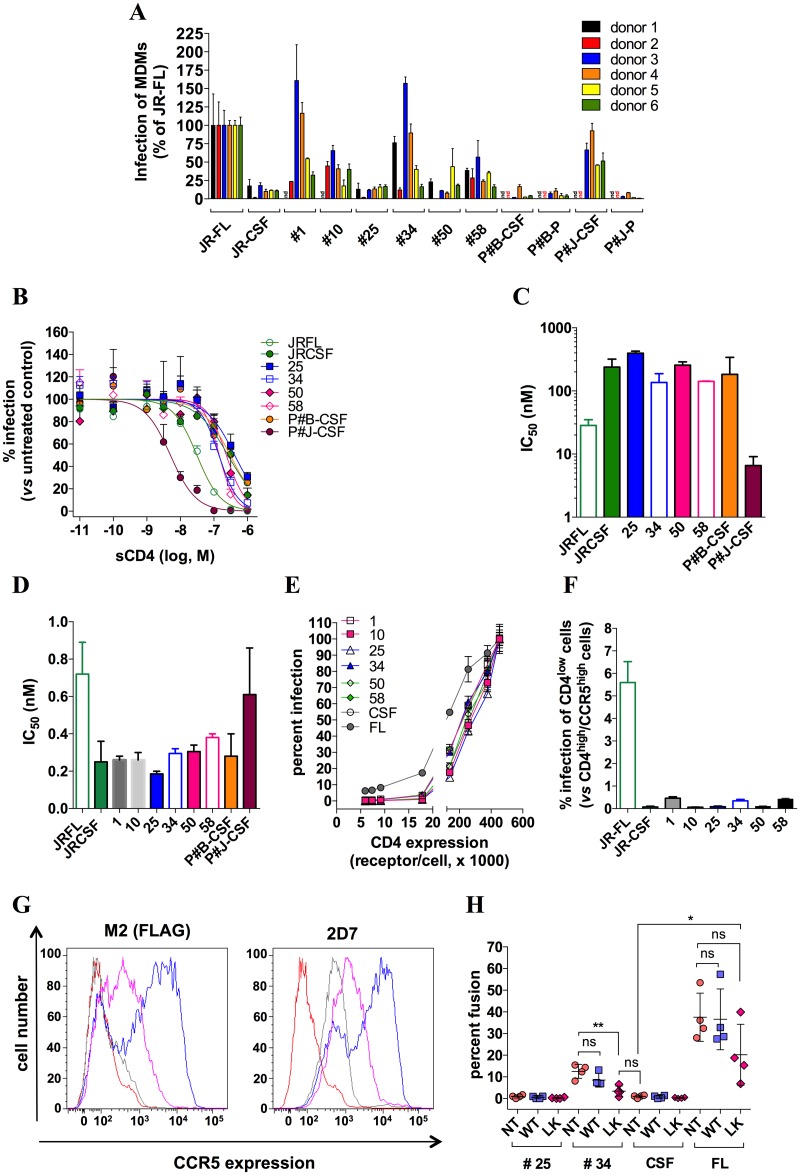
Blood-derived HIV-1 isolates depend on CCR5 plasticity for infection of MDMs. **A** MDMs from 6 different healthy donors were infected by virus clones pseudotyped with gp160 #1, #10, #25, #34, #50 or #58, the M-tropic or the non-M-tropic HIV-1 strain JR-FL or JR-CSF, respectively, or recombinant viral populations generated from plasma (P) or cerebrospinal fluid (CSF) of two patients with HIV-1-associated encephalitis (P#B and P#J). Virus quantities that produced comparable levels of infectivity in T-cells isolated from the same donors (*i*.*e*. 10^5^ RLU) were used. Of note, similar results were obtained after normalizing the virus quantities to the p24 content. Results represent infectivities of the viruses in MDMs, determined in duplicate, and normalized to infectivity of JR-FL (arbitrarily set at 100%). **B** Sensitivity of viruses to sCD4. Some of the previous viruses were tested for their sensitivity to inhibition by increasing concentrations of sCD4 in infection experiments of PHA/IL-2- activated CD4 T-cells. The virus amounts were selected in such a way that infectivities in the absence of sCD4 were similar (*i*.*e*. 10^5^ RLU of luciferase activity). The data points shown are from one representative experiment performed in duplicate, out of three experiments performed independently. The independent experiments were carried out with the lymphocytes from different healthy donors. Infectivity of the viruses was expressed as percentage of that measured in the absence of sCD4 (100%). Inhibition curves were fitted according to a sigmoidal dose-response model with a variable slope. **C** The panel shows sCD4 IC_50_ values that were deduced from inhibition curves with GraphPad Prism 6. Results are means ± SD of 3 independent determinations. **D** Dose-response inhibitions of infection of activated CD4 T-cells by increasing concentrations (ranging between 10^−12^ and 3x10^-8^ nM) of the anti-CD4 mAb Q4120 were carried out with the indicated viruses. Then, IC_50_ values for inhibition of infection by Q4120 were calculated with GraphPad Prism 6. The data shown here represent means ± SEM of two independent experiments performed in duplicate. **E** Infection assays of Affinofile cells expressing CCR5 at a high level (≈ 10^5^ receptors/cell, stimulated by 2 μM ponasterone A) and increasing amounts of CD4 stimulated by 7 different concentrations of minocycline ranging between 0.08 and 2.5 ng/ml (see “Materials and methods” for further details). Data points represent infectivities of viruses that were normalized to infectivity in Affinofile cells expressing the highest levels of CCR5 and CD4 (CD4^high^/CCR5^high^ Affinofile cells), arbitrarily taken as 100%. One representative experiment out of three independent experiments performed in duplicate is shown. In those experiments, the quantities of the different viruses were adjusted in such a way that they generated similar RLU in CD4^high^/CCR5^high^ Affinofile cells (10^5^). However, similar results were also obtained when normalizing the quantities of viruses to the p24 content. **F** Infectivities of the indicated pseudotyped viruses in CD4^low^/CCR5^high^ affinofile cells (cells that have not been stimulated by minocycline, and where the CD4 expression level is minimal, *i*.*e*. in the range of 4000 receptors/cell), expressed as percentage of the infectivity in CD4^high^/CCR5^high^ Affinofile cells). **G** Receptor expression levels at the surface of untransduced (grey line) or transduced MDMs with pTRIP ΔU3-expressing FLAG-tagged WT-CCR5 (blue line) or L196K-CCR5 (magenta line). Results were obtained using the anti-Flag mAb M2 (left panel) or the anti-CCR5 mAb 2D7 (right panel) revealed by an AlexaFluor 647-conjugated goat anti-mouse IgG (GAM) and flow cytometry analysis. With the mAb M2, GMFI values equal to 7836 and 2010 were found for WT-CCR5 and L196K-CCR5. Background signal of untransduced MDMs labeled with GAM alone is represented as red lines. **H** Levels of fusion of BlaM-vpr-containing virus #25, virus #34, JR-CSF or JR-FL to untransduced or transduced MDMs. Fusion was measured by flow cytometry analysis 3 h post-inoculation, as described in the “Materials and Methods” section. Each data point represents the level of fusion of the indicated virus clone with MDMs from one donor, expressed as a percentage of MDMs where CCF2 is cleaved by BlaM. On the whole, four independent experiments with MDMs from four healthy individuals were done. The panel displays the means +/- SD of the results obtained from these 4 experiments.

As part of the present study, we sought to investigate whether the nature of the CCR5 forms that are present on macrophages, which differ from those on T-cells (S2 Fig and ref. [14]), influences HIV-1 entry into macrophages. We wondered whether altering CCR5 dimerization, similarly to what we have just described in T-cells (Fig 5), could be likely to modulate efficiency of entry into macrophages. To this end, we first assessed the degree to which the virus clones pseudotyped with our Envs infect MDMs (Fig 6A). Infectivity of the viruses was compared to that of the M-tropic virus JR-FL (arbitrarily set at 100%) and the non M-tropic virus JR-CSF. In an effort to give the best picture of M-tropism of our Envs, two additional controls were done. First, as sensitivity of MDMs to HIV-1 infection can vary greatly between different donors [63], experiments were repeated with MDMs from 6 different donors (Fig 6A). Second, recombinant viral populations from plasma and CSF of two infected patients (Patients #B and #J) with HIV-associated encephalitis were also included in the experiments. The virus population in the CSF of P#J (P#J-CSF) declined only very slowly under cART (see “Materials and methods”), suggesting that these viruses are derived from long-lived cells. In accordance with this, the viruses consistently infected MDMs in our hands, similarly to JR-FL (Fig 6A). In contrast, none of the virus populations from the other patient’s samples (P#B’s plasma/CSF and P#J’s plasma), which were observed to decay rapidly under cART, could do so (similarly to JR-CSF). Thus, we confirm here the link drawn before between cellular tropism of HIV-1 and response of patients to cART [60, 65].

Regarding their capacity to infect MDMs, the Env-pseudotyped virus clones fell in two groups. Virus #25, the only representative from the first group among the viruses we tested, behaved similarly to JR-CSF and consistently failed to infect MDMs, while maintaining robust infection of T-cells (Fig 6A). Most of the other viruses however could infect MDMs, but this greatly varied upon the nature of the donor. This is exemplified for virus #34 that infected MDMs from donors 1, 3 and 4 as efficiently as JR-FL and P#J-CSF, but did so more weakly with donors 2 and 5. However, infection of MDMs from donor 6 by virus #34 did not differ from that by virus #25 or JR-CSF. On the whole, infectivity of JR-FL and P#J-CSF was less affected by the nature of the donor, as compared to that of the Env-pseudotyped virus clones. The results in Fig 6A further show that the distinct pseudotyped virus clones were differentially influenced by the nature of the MDMs (*e*.*g*. virus #34 infected better MDMs from donors 3 and 4 than virus #10, while the reverse was observed with donors 2 and 6).

Several lines of evidence demonstrated that although the Env-pseudotyped viruses can infect MDMs in some circumstances, and even very efficiently in some cases, they differ from the prototypical M-tropic viruses JR-FL and P#J-CSF in that they don’t show increased affinity for CD4. Indeed, while JR-FL and even more P#J-CSF had increased sensitivity to sCD4, as expected for “true” M-tropic strains [43, 49, 59, 62], this was not the case for the other viruses. The IC_50_ values for inhibition of the Env-pseudotyped virus clones by sCD4 did not differ from those of the non M-tropic viruses JR-CSF, P#J-plasma and P#B-CSF/plasma. They all lay in a range of few hundreds of nM, thus about one-log higher than those of the M-tropic viruses (Fig 6B and 6C), as previously reported [43, 59]. Accordingly, the pseudotyped virus clones and JR-CSF were also more sensitive to inhibition by the anti-CD4 mAb Q4120, as compared to the M-tropic viruses (Fig 6D and [43]). Last, we compared the virus clones to JR-FL and JR-CSF in infection assays of Affinofile cells expressing high levels of CCR5 and increasing amounts of CD4, as previously reported [62, 63]. As shown in Fig 6E and 6F, while JR-FL could infect Affinofile cells at the lowest CD4 expression levels, in agreement with recent data [66], the other viruses required higher CD4 expression levels. Considered altogether, these results demonstrate that the Envs studied here are not adapted to infection of cells expressing low levels of CD4. As such, they resemble more the R5 T-tropic strains that predominate in the blood of patients than the “true” R5 M-tropic viruses found in brain tissue. Actually, this is in accordance with the blood origin of the primary viruses from which these Envs were isolated. Thus, even if some of the Env-pseudotyped virus clones can infect MDMs in some cases, this cannot be attributed to increased efficiency of CD4 usage.

We next reasoned that differences in the mode of CCR5 usage could distinguish between those pseudotyped viruses that can infect MDMs in some cases (*e*.g. virus #34) and those that cannot do so (*e*.*g*. virus #25). In particular, in light of the results obtained in T-cells, we asked the question of whether impairing CCR5 dimerization could promote entry into MDMs. To test this, we expressed by lentiviral transduction WT-CCR5 or L196K-CCR5 in freshly prepared monocytes, and then differentiated these cells into MDMs for 7 days. The mean receptor expression levels (detected by the anti-CCR5 mAb 2D7) increased by up to 10- and 3-fold in WT-CCR5- and L196K-CCR5-expressing MDMs, respectively, as compared to untransduced MDMs (Fig 6G), and were in the same range as those measured in transduced A3. 01 T cells (see the legends of Figs 5B and 6G). We then measured fusion of virus #25, #34, JR-CSF or JR-FL with untransduced or transduced MDMs. JR-FL and to a reduced extent virus #34, but only marginally virus #25 and JR-CSF, could fuse with MDMs (Fig 6H). In these cells, however, the impact of the receptors on viral entry differed markedly to that in T-cells. Notably, the extent of fusion of the viruses with WT-CCR5-overexpressing cells was similar to that with untransduced (NT) MDMs. This result indicates that HIV-1 entry into MDMs, in contrast to T-cells (Fig 5C, 5D and 5E), hardly depends on the overall CCR5 expression level, regardless of whether or not the viral isolate uses CD4 efficiently. We also observed that L196K-CCR5 substantially diminishes the ability of virus #34 to fuse with MDMs, in contrast to the effect it has in T-cells, indicating that this receptor has a reduced capacity to support HIV entry into MDMs, as compared to T-cells. A similar trend was observed for JR-FL, albeit not significant. This result, together with our data in S2B and S2C Fig showing that CCR5 has gp120 binding capacities that differ between T-cells and MDMs, indicate that the capacity of CCR5 to function as a HIV coreceptor may differ between different cell types and this is likely to contribute to modulate sensitivity of target cells to HIV infection. However, our results suggest that this effect of CCR5 is less marked in the case of viruses that use CD4 efficiently (JR-FL).

## Discussion

Different aspects of the pathogenesis of HIV infection have been interpreted in light of the properties of HIV/receptor interactions. Alteration in the affinity of R5 Envs for CD4 and/or CCR5 has been proposed to influence the virulence of R5 viruses, HIV resistance to entry inhibitors and cell tropism [40, 43, 48, 64, 67–69]. However, none of these studies has considered that CCR5 exists in structurally different forms, the multiplicity of the modes of gp120/CCR5 interactions and the possibility that distinct HIV Envs differ in the nature of the CCR5 populations to which they bind. We show here that all of these factors are likely to shape phenotypic properties of viruses and their role in infection.

Here, distinct gp120s display divergent binding levels to CCR5-expressing cell lines and primary cells (Fig 1 and S2 Fig). This is partly explained by the fact that different gp120s can bind differentially antigenically distinct populations of CCR5 (S3 Fig), which exist in different quantities in cells [11, 14]. This also owes to distinct capacities of gp120s to bind to CCR5 dimers (or higher order oligomers) (Fig 3), which can themselves also exist in different conformations [52]. Some gp120s (e.g. gp120 #25 and #34) also compete each other for binding to the same receptors (Fig 2), while showing great differences in their binding levels to CCR5. This apparent paradox could be explained by a model whereby the gp120s engage the CCR5 dimers with distinct stoichiometries (Fig 4). Our data indicate that the different CCR5 populations and how they bind the gp120s vary according to the conformation of the receptor ECL2. The L196K mutation in TM5 to which ECL2 is connected alters accessibility of the loop to mAbs and differentially changes the binding levels of the different gp120s (Fig 3E and 3F). Moreover, minimal change in the composition of the tip of the gp120 V3 loop, which interacts with ECL2, modifies the binding capacities of gp120s (Fig 3E) [32, 42]. The gp120s used here contain sequence changes in V3 and other CCR5 binding regions (S7 Fig), which similarly could determine which population(s) of CCR5 they recognize.

Here, we show that HIV-1 gp120s/isolates and anti-CCR5 mAbs do not necessarily recognize the same CCR5 conformations, explaining why anti-CCR5 mAbs can be inefficient as HIV-1 entry inhibitor (S3 Fig). Other CCR5 ligands target particular CCR5 conformations, which in some cases may differ from those used by viruses [27, 70, 71]. We recently identified that R5 HIV-1 strains escape inhibition by CCR5 chemokines by exploiting low-chemokine affinity conformations of CCR5, which are uncoupled from nucleotide-free G proteins [12, 16]. This is due to the fact that HIV-1 gp120s, in contrast to chemokines, bind CCR5 independently of its coupling to G proteins. Here, we provide further evidence that CCR5 exhibits differential conformational requirements for binding chemokines and gp120. This is illustrated by the differential effects of the L196K mutation on CCL3 and gp120 binding (Fig 3). The decrease of CCL3 binding to L196K-CCR5, which forms less dimers than the wild-type receptor (Fig 3B and [52]), suggests that CCR5 dimerization could be required for high affinity binding of agonist chemokines to the receptor. This suggests that a link could exist between CCR5 dimerization and its coupling to G proteins, although data on other GPCRs favor the idea of a 1:1 interaction stoichiometry between activated receptors and G proteins [72–75]. Previous work already suggested that CCR5 homodimers are involved in high affinity binding of chemokines in a G-protein dependent manner [20]. Alternatively, the proportion of receptors that exist in a high-affinity conformation for CCL3 could be decreased for L196K-CCR5, regardless of its dimerization state. Whichever the mechanism that is involved in the decrease of CCL3 binding to L196K-CCR5, we show here that expression of this mutant results in increase of HIV-1 entry into T-cells (Fig 5). This again evidences that HIV-1 and CCR5 chemokines recognize distinct conformations of the coreceptor.

Here, we illustrate that the number of CCR5 molecules to which gp120s is likely to bind may influence viral infectivity (Fig 5). This parameter could therefore act in concert with HIV-1 entry stoichiometry T, *i*.*e*. the number of Envs required for entry, which varies between different HIV-1 strains and also regulates efficiency of viral entry [31, 76]. As the relative proportions and the nature of the CCR5 forms vary from one cell type to another (S2 Fig) [11, 14], recognition of a wide variety of the CCR5 forms could also allow a virus to have an extended cell tropism. This could increase its chances of infecting cells that express limiting amounts of the receptor and where, for this reason, CCR5 diversity could be narrower, such as in naïve and central memory T cells (Tcm) whose loss in the late stages of infection is thought to contribute to development of AIDS [77].

Two observations suggest that monomeric gp120s and complete viruses can show differences in the nature of the CCR5 forms they target. First, some anti-CCR5 mAbs that weakly displace gp120 binding (*e*.*g*. 2D7) are efficient entry inhibitors, and conversely for other mAbs (S3 Fig). Second, the level of gp120 binding measured by means of binding assays is not necessarily correlated with viral entry capacity (our results related to gp120 #10 and 58 in Fig 1A and ref. [42, 78]). We do not believe that these data could owe to the fact that virus-associated gp120s and monomeric gp120s stabilize distinct conformations of the coreceptor. This would imply that these two forms of the gp120 differ in the conformation of their coreceptor-binding regions, which is not supported by the available structural and functional data. Indeed, in the CD4-bound, open form of the Env trimer [79], the three gp120 protomers are held separate, and adopt a conformation of their core and coreceptor-binding regions that is very close to those in soluble gp120 monomers [34, 35, 80]. In line with this, comparison studies have shown that virus- (or membrane)-associated gp120 and soluble, monomeric gp120 induce CCR5 signaling effects that are largely comparable [81–84]. This strongly suggests that complete viruses and monomeric gp120 stabilize the same active conformations of the coreceptor.

We believe it more likely that viruses and gp120s bind similar CCR5 forms, but only a subset of them is used for viral entry. This is consistent with the view that CCR5 requires distinct molecular determinants for binding gp120 and mediating fusion [85, 86], and with our recent observation that viruses can bind to CCR5 with high affinity without necessarily entering into cells [87]. Our results here suggest that CCR5 homodimers could represent a form of the coreceptor that allows gp120 binding but not entry. Indeed, while results in Fig 3 suggest that gp120s bind CCR5 monomers and oligomers with similar affinity, the infection assays in Fig 5 are consistent with viruses using predominantly CCR5 monomers for entry, in agreement with previous works [88, 89]. Besides that, dichotomies between the gp120 binding and infection assays could simply relate to the fact that some CCR5 receptors to which sCD4-gp120 can bind do not satisfy critical conditions for viral entry, such as co-localization with CD4 and/or be surrounded by membrane lipids that are appropriate for fusion [90, 91].

CCR5 differs in its capacity to bind gp120 (S2B and S2C Fig) and to mediate viral entry (Figs 5 and 6) between T-cells and MDMs. This could owe to the fact that CCR5 does not adopt the same conformations in T-cells and MDMs [14]. Our results suggest that CCR5 also exhibits different organization at the surface of both cell types. Indeed, MDMs and T-cells express similar quantities of CCR5 (legend of S2 Fig), but MDMs have a lower CD4 density [63]. In this situation, it seems paradoxical that viral entry depends on the CCR5 expression level in T-cells (Fig 5), but not in MDMs (Fig 6G and 6H and [92]). This could indicate low efficiency of CCR5 usage in MDMs. Indeed, the HIV-1 strain 89.6 binds CCR5 very weakly [85] and is similarly not influenced by the CCR5 expression level in Affinofile cells, regardless of the CD4 expression level [93]. This could also reflect that CCR5 in MDMs is clustered in membrane subdomains where its density is already at a saturating level. Actually, HIV-1 entry and replication increase with CCR5 density [94–96], and this is what we observed in T-cells (Fig 5). But increase in density of GPCRs in a certain range also increases the proportion of these receptors that exist in dimers/oligomers [53]. Although hypothetical at this time, a higher proportion of CCR5 dimers in MDMs would explain why gp120 # 25 binds less efficiently those cells than T-cells (S2 Fig). This would also explain why L196K-CCR5 decreased HIV-1 entry into MDMs, (Fig 6H), while it increased it into T-cells (Fig 5). In T-cells, results are compatible with the view that the proportion of L196K-CCR5 that exists as monomers is higher compared to WT-CCR5. This however might not be the case in MDMs in the context of high receptor density. Under conditions where the receptor density is high, heterodimers between L196K-CCR5 and endogenous CCR5 could also form in MDMs in addition to receptor monomers. These heterodimers could adopt a conformation that is not competent for viral entry. This hypothesis is actually consistent with the prevalent view that GPCRs have distinct structural and functional properties depending on whether they are engaged in homo- or hetero-dimers ([97, 98] and for review [5]).

Most of R5 HIV-1 strains are less efficient at entering MDMs than T-cells [56]. This is argued to result from low CD4 density in MDMs [63], but low efficiency of CCR5 usage could also contribute to this phenotype. Viruses adapted to infect macrophages are frequent in brain tissue [61]. These viruses were shown to use CD4 very efficiently [43, 48, 49, 60, 62, 63]. In some studies, however, M-tropism has been attributed to changes in the binding characteristics to CCR5 [64, 67]. Viruses with phenotypic properties resembling those of M-tropic viruses from brain are rare in blood and immune tissues [56]. However, we identified here that Envs from biological virus clones of patient’s PBMCs could infect MDMs, although this hugely depended upon the nature of the donor (Fig 6). Previous works have emphasized that long-term culturing of HIV-1 isolates on PBMCs or T-cells favors acquisition of phenotypic traits of M-tropic viruses (e.g. increased sensitivity to sCD4, resistance to anti-CD4 mAbs, and ability to infect cells expressing low levels of CD4 [66, 99, 100]), presumably due to stabilization of a partially opened conformation of Env [66, 101]. The Envs studied here, however, do not show such functional characteristics (Fig 6). They also lack genetic traits of virus adaptation to long-term culture on PBMCs [66], such as mutation of Leu193 in the V2 loop, a residue that maintains the Env trimer in a closed conformation. Other residue changes that feature culture-adapted viruses [66, 99, 100] are absent or present only sporadically in the sequences of our Envs (S7 Fig). Most of the Envs also lack the genetic signatures that have been associated to M-tropism, such as T283N [48], E153G [102] and N386D [103]. There are some exceptions, such as in the sequences of gp120 #34 and #58, where the T283N is present. An Asn residue at position 283 has been observed to confer increased affinity for CD4 and improve macrophage tropism [48]. Substitution of Asn by a Thr residue in the sequences of gp120 #34 and #58 however did not change affinity for CD4 [41], suggesting that the effect of the T283N mutation is context-dependent.

The Env-pseudotyped viruses we used here thus do not show increased efficiency of CD4 usage (Fig 6) and as such, share phenotypic characteristics of R5 T-tropic viruses isolated from blood [49, 59, 62]. Similarly to our viruses, other T-tropic viruses have been reported to infect macrophages, albeit in most of the cases to a lower level as compared to “true” M-tropic viruses [58, 60, 63, 64]. However, in agreement with previous data [63], we show here that the degree to which T-tropic viruses support infection of MDMs is a relative concept that actually greatly depends upon the nature of the donor. This suggests that key parameters that control infection of MDMs by R5 T-tropic viruses are differentially expressed from one type of MDMs to another. Our results in Fig 6 show that CCR5 structural plasticity dramatically regulates entry into MDMs of a R5 T-tropic virus (virus #34), while it has a weaker effect on entry of the M-tropic strain JRFL that uses CD4 efficiently. It has been reported that the CCR5 populations on MDMs differ between different donors [14], and in light of the results shown here, it is tempting to speculate that this could contribute to the fact that MDMs from different donors are differentially sensitive to infection by R5 T-tropic viruses (although we cannot exclude that post-entry steps could also be involved [54]). Our study also raises the possibility that some R5 T-tropic viruses could contribute to the first steps of the colonization of the central nervous system (CNS) in HIV+ patients. Indeed, it has been shown that this process occurs early in infection and is mediated by infected CD4+ T-cells [104]. Baseline replication in macrophages/microglia of R5 T-tropic viruses thanks to their use of particular CCR5 conformations could precede adaptation to usage of low CD4 levels. In this context, the nature of the CCR5 population expressed on macrophages/microglia, if it turned out that it actually regulates the differences in their sensitivity to infection by R5 T-tropic viruses between different individuals, would represent a risk factor to infection of the CNS.

## Materials and methods

### Cells, reagents and plasmids

The A3.01 human T-cell line (obtained from Dr H-T He, Centre d’Immunologie INSERM/CNRS de Marseille-Luminy, France), HEK 293T cells (obtained from American Type Culture Collection (ATCC)) stably expressing or not (parental cells) CCR5 (HEK-R5) or CD4 (HEK-CD4), and the HEK 293 cells (obtained from ATCC) expressing FLAG/SNAP-tagged WT- or L196K-CCR5, were described previously [12, 16, 42]. U87-R5 cells were generated by transducing the human glioblastoma cell line U87 (obtained from ATCC) with the pTRIP ΔU3 lentiviral vector (a gift from Dr P. Charneau, Institut Pasteur, Paris) encoding the CCR5 sequence. These cells were cultured in Dulbecco’s Modified Eagle Medium (DMEM) supplemented with 10% (v/v) foetal bovine serum (FBS), 100 μg/ml streptomycin and 100 units/ml penicillin. The 293-Affinofile cell line (a gift from Dr B. Lee, Mont Sinai Hospital, New York, NY, USA) was maintained in DMEM supplemented with 10% (v/v) foetal bovine serum, 100 μg/ml streptomycin, 100 units/ml penicillin, and 50 μg/ml blasticidin (Invitrogen). Human CD4+ T-lymphocytes and CD14+ monocytes were purified from Peripheral Blood Mononuclear Cells (PBMC) of healthy blood donors from Etablissement Français du Sang (EFS, The French Official Blood Bank) in accordance with the EFS ethical guidelines by Ficoll centrifugation (PAA laboratories) and subsequent immunomagnetic positive selection using CD4 or CD14 MicroBeads (Miltenyi Biotec). CD4+ T-cells were maintained for 2 days in RPMI 1640 medium containing recombinant interleukin-2 (IL-2) (300 IU/ml) and phytohemagglutinin (1 μg/ml, Thermo Fisher Scientific), and then for additional 4–6 days in the presence of IL-2 alone. CD14^+^ monocytes were differentiated into macrophages in M-SFM medium (Thermo Fisher Scientific) containing 50 ng/ml M-CSF (Miltenyi Biotec), penicillin (100 U/ml) and streptomycin (100 μg/ml) for 7 days.

Recombinant soluble human CD4 (sCD4) was produced and purified as previously described [42]. The anti-CCR5 mAbs CTC5, 2D7 and 45531 were obtained from BD Biosciences and R&D Systems. The unconjugated and AlexaFluor 647-conjugated forms of the anti-Flag mAb M2 were obtained from Sigma-Aldrich and Cell Signaling Technologies, respectively. The anti-CD4 mAb Q4120 was described in our previous work [42]. The goat anti-mouse AlexaFluor 647-conjugated secondary antibody was from Invitrogen. MAb E51 and Maraviroc were obtained from the AIDS Research and Reference Reagent Program.

### Ethics statement

Blood samples from anonymous healthy donors were obtained from Etablissement Français du Sang (EFS, the French National Blood Agency). Sample use for scientific purpose was carried out in accordance with convention between EFS and Institut Pasteur. All donors have provided written informed consent at the time of blood collection. Some of the envelope glycoproteins studied here (*v*.*i*.) were isolated from virus clones previously described in references [105, 106]. These viruses were kindly provided to us by Pr.Dr. H. Schuitemaker (University of Amsterdam, The Netherlands), who isolated them from PBMCs of anonymized participants of the Amsterdam Cohort Studies (ACS) on HIV-1 and AIDS. The ACS is a well-reported cohort study (https://www.amsterdamcohortstudies.org) that started in 1984 and enrolled HIV-1 infected and HIV-1-uninfected individuals at high risk for HIV infection. The ACS obeys the principles expressed in the Declaration of Helsinki and was approved by the Medical Ethics Committee of the Academic Medical Center in Amsterdam. Participants were volonteers who provided written informed consents for blood usage in research investigations.

The patients #B (P#B) and #J (P#J), to whom reference is made here, attend the Infectious Diseases Department of Toulouse University-Hospital, France, for care and have provided informed written consent for research investigations on their clinical samples. P#B was diagnosed with HIV-1 in 1993 and further developed clinical, CSF, and brain MRI patterns supporting the diagnosis of HIV encephalitis in 2017. At that time, HIV-1 viral loads (VLs) in the plasma and CSF of P#B attained 2.74 and 4.31 log_10_ copies/ml, respectively. R5 tropism was confirmed genotypically and phenotypically for plasma and CSF viruses. The modification of cART regimen with Tenofovir, Emtricitabine, Darunavir/Ritonavir, and Maraviroc led to robust decrease of VLs in both plasma and CSF within the following seven months up to values below the detection threshold. P#J was diagnosed with HIV-1 infection in 2017. At this time, P#J exhibited a marked depletion in blood CD4 T-lymphocytes (< 100 cell/mm^3^) and severe clinical manifestations of HIV-associated dementia with brain MRI abnormalities. Measurement of HIV-1 RNA in the plasma and CSF of P#J revealed VLs of 6.26 and 4.44 log_10_ copies/ml, respectively. R5 tropism was confirmed genotypically and phenotypically for plasma and CSF viruses. P#J first received cART with Abacavir, Lamivudine, and Dolutegravir. Genotypic drug-resistance profiles in plasma and CSF confirmed full sensitivity to these drugs. Over the first three months of treatment, the VL in the plasma considerably felt down, up to a value of 1.85 log_10_ copies/ml, in contrast to the VL in the CSF that did not change (4.35 log_10_ copies/ml). cART treatment was optimized for CNS penetration with Abacavir, Lamivudine, Nevirapine, and Maraviroc. After 5 months of cART, the VL in the CSF still remained high (4.05 log_10_ copies/ml), while that in the plasma continued decreasing (1.54 log_10_ copies/ml). At that time, HIV drug-resistance testing was repeated and confirmed that the viruses in the CSF are genotypically drug-sensitive.

### HIV-1 envelope glycoproteins: Description, production and purification

The sequences coding for envelope glycoproteins #1, 10, 25, 34, 38, 48, 50, 58 and 59 (displayed in S7 Fig) were isolated from R5-tropic biological virus clones #15.3A7, 46.5B1, 14.5C6, 75.6C4, 12.C12, 43.5D3, 20.8C1, 65.9D8, and 65.9E7, respectively. These clones were isolated from anonymized individuals of the ACS who were previously identified as H1 (Env #1,10), H2 (Env #38,48), H3 (Env #50,58, 59) and H5 (Env #25,34) in references [105, 106] and were recoded here as patients #1, 458, 1031 and 341, respectively. PBMCs were collected early after seroconversion (SC) (Env #1, 25, 38, and 50 were collected 26, 30, 20 and 22 months after SC, respectively) or in the AIDS stage of infection (Env #10, 34, 48, 58, and 59 were collected 107, 128, 86, 91 and 91 months after SC, respectively). The mean numbers of CD4+ T-cells in the blood of patients were 640 and 90 per μl at the early and late stages of infection, respectively. The envelope glycoprotein from the HIV-1 primary strain Bx08 was previously described [9]. Envs # 1f and 1f(V3) correspond to envelope glycoproteins MVC-Sens and MVC-Sens(P/S) described in our previous work [42]. Sequences for the laboratory-adapted HIV-1 strains JR-CSF and JR-FL can be found in the Los Alamos HIV sequence Database (https://www.hiv.lanl.gov/).

Extraction of HIV-1 viral RNA from PBMCs, amplification of *env* gene sequences, cloning of gp120 sequences in the Semliki Forest Virus-derived expression vector pSFV2, and production, ^35^S labelling and purification of soluble, monomeric gp120 fused to the One STrEP-tag (IBA GmbH) at their C-tail have recently been described in detail [41]. The sequences encoding the different full-length envelope glycoproteins (gp160) were synthesized and cloned in the pNL-SacII-lacZ/env-Ren proviral vector in collaboration with the Genscript Company. This vector derives from the HIV-1 proviral clone pNL4-3Ren [107], in which the *nef* gene in the genome of the HIV-1 strain NL4-3 is replaced by the *Renilla luciferase* gene at the NotI site in position 8797, and was constructed similarly as to the pNL-lacz/env-Ren proviral vector previously described [108]. Briefly, pNL-SacII-lacZ/env-Ren was generated by introducing a SacII site at position 6113 in pNL4-3Ren and replacing the *env* coding sequence between SacII (6113) and NotI (8797) by the amino-terminal fragment of the *lacZ* gene. Then, the fragment in pNL-SacII-lacZ/env-Ren between EcoRI (5743) and NotI was deleted and replaced by the *env* genes flanked at their 5’ and 3’ ends by the sequences of NL4-3 between EcoRI/SacII and upstream the NotI site, respectively.

### Preparation of virus clones and recombinant virus populations

The Env-pseudotyped virus clones described here were produced by transfecting HEK 293T cells with the *env*-containing pNL-SacII-lacZ/env-Ren proviral vectors, as previously described [87]. For preparation of the recombinant virus populations from the plasma and CSF of patients P#J and P#B, the *env* sequence region encompassing gp120 and ectodomain of gp41 was amplified and then cotransfected with NheI-linearized pNL43-Δenv-Luc2 vector DNA in HEK 293T cells, as described [109]. Forty eight hours post-transfection, cell culture supernatants were harvested, clarified by centrifugation and frozen at—80 °C. The amount of p24 antigen in the supernatants was quantified using a commercially available ELISA kit (Innotest HIV antigen mAb; Innogenetics, Gent, Belgium). All of the viruses used here (including the virus populations from P#J and P#B) were confirmed to be R5-tropic in phenotypic assays using CCR5- or CXCR4-expressing U87-CD4 cells as indicator cells.

### Lentivirus production and cell transductions

Lentiviral particles expressing SNAP/FLAG tagged WT-CCR5 or L196K-CCR5 were produced in HEK 293TN cells. Cells were cotransfected using lipofectamine 3000 (Invitrogen) with the plasmid pTRIP ΔU3-CMV containing either of the two receptor sequences, the encapsidation plasmid p8.74, the vesicular stomatitis virus G protein-expressing plasmid pVSV-G and the HIV-1 Rev protein-expressing plasmid pRev at a 3:2:1:1 ratio (The plasmids were kindly provided by Dr P. Charneau, Institut Pasteur, Paris). Forty-eight hours post-transfection, culture supernatants were collected, cleared by centrifugation at low speed and filtration (0.45 μm) and ultracentrifuged at 4°C for 120 min at 83000 x g. The amount of VSV-G-pseudotyped lentiviral particles was determined by measuring the Gag p24 concentration with the Alliance HIV-1 p24 Antigen ELISA kit (Perkin Elmer).

For transduction of A3.01 T cells, 0.5 x 10^6^ cells were incubated for 72 h with 25 or 400 ng Gag p24 of WT-CCR5- or L196K-CCR5-expressing lentiviral particles, respectively. Receptor-expressing cells were then washed, kept in culture for several days and then frozen in liquid nitrogen before use. For expression of receptors in MDMs, lentiviral transductions were carried out as follows. Sorted CD14+ monocytes were plated on 6-well plates (2 x 10^6^ / well) and then transduced with 150 or 450 ng Gag p24 of SNAP-FLAG tagged WT-CCR5- or L196K-CCR5-expressing lentiviral particles in the presence of Vpx-expressing SIVmac 251-derived particles produced using the pSIV3+ plasmid (a kind gift from Dr O. Schwartz, Institut Pasteur, Paris). Transduced monocytes were then allowed to differentiate into macrophages as described above for 7 days before being used in the fusion assays.

### Binding experiments

Most of the protocols for the binding assays used herein have already been detailed in a step-by-step manner in our previous articles to which readers may refer, including a recently published methodological article [41]. Equilibrium binding of ^35^S-gp120 or ^125^I-CCL3 to intact cell lines or membrane preparations from these cells was measured and analyzed as described in references [9, 41, 42]. Scatchard transformation of data from equilibrium saturation binding experiments of ^35^S-gp120 to HEK-R5 membranes was performed with GraphPad Prism 6. Binding of 10 nM ^35^S-gp120 to 1.5 x 10^6^ activated CD4+ T-cells or 1 x 10^6^ MDMs was carried out similarly in the presence of 300 nM sCD4. Crude membrane preparations from these cells were prepared as described previously [10], and 20 μg (T-cells) or 40 μg (MDMs) of membrane proteins were used in the binding assays. The protocol for the displacement experiments of ^35^S-gp120 #34 binding to HEK-R5 membranes by increasing concentrations of unlabeled gp120 is the same as that described in reference [42]. The displacement experiments of Q4120 binding to HEK-CD4 cells by gp120s have recently been detailed and commented extensively [41]. Finally, surface plasmon resonance analysis of the interaction between gp120 and mAb E51, in the presence or absence of 200 nM sCD4, was performed similarly as in our previous work [42].

### Receptor immunostaining and flow cytometry analysis

For assessment of receptor expression levels by flow cytometry, cells (2 × 10^5^) in conical-bottom 96-well plates were incubated for 1 h at 4°C in 0.1 ml final volume of FACS buffer (phosphate-buffered saline (PBS), 1% BSA, 0.1% NaN_3_) containing unconjugated anti-Flag mAb M2 (usually 2 μg/ml) or anti-CCR5 mAb 2D7 (2.5 μg/ml). In Fig 3F, the concentrations of unconjugated mAbs (M2, 2D7 and 45531) were saturating (40 μg/ml). After two washing steps in ice-cold FACS buffer, cells were incubated at 4°C for 30 min in 0.1 ml FACS buffer containing AlexaFluor 647-conjugated goat anti-mouse IgG (Life Technologies) at a 1:500 dilution. Cells were then washed twice in the FACS buffer and fixed with 2% paraformaldehyde-containing PBS. Data were acquired out on a FACSCanto flow cytometer (BD Biosciences) and analyzed using FlowJo software. In the particular case of immunostaining of MDMs, 1.5 × 10^5^ cells in 50 μl of FACS buffer were first incubated for 1 h at 4°C in the presence of 10% human serum AB (hSAB). Cells were then further incubated at 4°C for 20 min in 30 μl of FACS buffer in the presence of FcR blocker (2 μl / 10^6^ cells, Myltenyi Biotec.). Then, labeling of receptors was carried out and analyzed as described above.

### TR-FRET measurements

Receptor homodimerization in HEK 293 cells was measured after cotransfection of cells with the FRET donor plasmid encoding FLAG/SNAP-tagged WT-CCR5 (20 ng), L208K-CCR5 (20 ng) or L196K-CCR5 (30 ng) and the FRET acceptor plasmid encoding FLAG/CLIP-tagged WT-CCR5 (10–20 ng), L208K-CCR5 (10–40 ng) or L196K-CCR5 (10–40 ng), respectively. These plasmid quantities were such that receptors showed similar expression levels at the cell surface and that the FRET acceptor(CLIP) / donor(SNAP) concentration ratio is non-saturating. Twenty-four hours post-transfection, the cells were detached, plated on black poly-D-lysine treated 96-well plates (Greiner), and incubated for a further 24 h. Cell surface expression of FLAG-SNAP- or FLAG-CLIP-tagged receptors was then determined by labeling cells (2 h, 37°C) either with 100 nM Snap-Lumi4Tb or 400 nM Clip-Lumi4Tb (Cisbio Bioassays, Codolet, France), exciting them at 320 nm and measuring fluorescence intensities at 620 nm. In separate wells, TR-FRET signals were obtained after colabeling cells with 100 nM Snap-Lumi4Tb and 400 nM Clip-d2 (Cisbio Bioassays, Codolet, France) (2 h, 37°C). After excitation at 320 nm, fluorescence emission intensities were measured at 665 nm for 400 μs after a 50 μs delay on a Mithras LB 940 (Berthold technologies). We calculated the net mTR-FRET ratio (*i*.e. FRET efficacy in Fig 3B) as (signal at 665 nm/signal at 620 nm) x 1,000 –background with donor plasmid alone.

### Single-round HIV-1 infection assays

For infection of U87-CD4-R5 cells, cells (2 x 10^5^ cells per well) in 96-well plates were inoculated with 100 ng p24 of the pNL4-3-derived viral clones expressing *Renilla* luciferase and gp160 from the JR-CSF or JR-FL strains, or the biological virus clones from patients of the ACS. Infected cells were further incubated at 37 °C for 48 h in complete culture medium (DMEM) in the presence or in the absence of CCR5 ligands (mAbs or MVC). Viral entry was then determined by measuring luciferase activity (Renilla Luciferase Assay, Promega, Madison, WI, USA) in the cell lysates using a Glomax luminometer (Promega). For infection of CD4 T-cells and MDMs, cells (2 x 10^5^ cells per well) in conical- (T-cells) or flat-bottom (MDMs) 96-well plates were incubated in complete RPMI medium supplemented with 20 ng/ml IL-2 (CD4 T-cells) or complete M-SFM medium containing M-CSF (10 ng/ml) (MDMs) in the presence or in the absence of entry inhibitors (Q4120 or MVC). For the inhibition assays by sCD4, viruses were first preincubated with the inhibitor for 45 min at 37 °C, as previously described [59]. Then, the virus-sCD4 mixtures were added to 2 x 10^5^ CD4 T-cells for additional 48 h at 37 °C. Infection experiments of Affinofile cells, as well as induction of CCR5 and CD4 expression by ponasterone A and minocycline, respectively, and determination of receptor expression levels (expressed as receptor number per cell), were carried out as described in our previous work [42]. The amounts of viruses used in the infection experiments of T-cells, MDMs and Affinofile cells were selected as described in the legends of Fig 6 (**panels A** and **E**). Infectivity was then determined by measuring Renilla (pNL4-3-derived viral clones) or Firefly (viral populations from P#B and P#J) luciferase activity (Promega, Madison, WI, USA).

### HIV-1-cell fusion experiments

BlaM-vpr-containing viral clones were prepared in HEK 293T cells as described [110]. Briefly, 1.5 x 10^7^ cells in 162 cm^2^ culture flasks were cotransfected using the calcium phosphate-DNA coprecipitation method with 60 μg proviral DNA, 20 μg pCMV-BlaM-vpr plasmid and 10 μg pAdvantage vector (Promega). Forty-eight hours post-transfection, culture supernatants containing the viral particles were clarified at low speed and then ultracentrifuged at 72,000 g for 90 min at 4°C. The pelleted viruses were then resuspended in DMEM, measured for their content in HIV-1 Gag p24 antigen (Alliance HIV P24 antigen ELISA Kit from PerkinElmer) and stored at -80 °C before use.

Fifty ng Gag p24 of BlaM-vpr-containing viruses were exposed to 2 x 10^5^ T-cells or 1.5 x 10^5^ MDMs, which were detached from culture flasks with Cellstripper (Corning), as previously described [42]. Then, cells were incubated for 2 h with the CCF2/AM dye (using the CCF2-AM loading kit from Invitrogen). Loading of the A3.01 T cells with CCF2 was performed in the presence of the AlexaFluor 647-conjugated anti-Flag mAb M2 at a 1:100 final dilution. Enzymatic cleavage of CCF2 by β-lactamase in the target cells was analyzed by flow cytometry (FACSCanto, BD Biosciences).

### Statistical analysis

The *t*- and *F*-tests presented in the study were realized using the GraphPad Prism software.

## Supporting information

S1 Table Binding parameters of the different HIV-1 gp120s used in the study. The K_D_ values represent the equilibrium dissociation constants of the ^35^S-gp120-sCD4/CCR5 complexes deduced in the saturation binding experiments to membranes from HEK 293T cells expressing CCR5 (HEK-R5 cells). The B_max_ values represent the maximum numbers of receptors binding the ^35^S-gp120-sCD4 complexes in these experiments. K_I_^Q4120^ and K_I_^gp120 #34^ represent the equilibrium dissociation constants for interaction of the indicated unlabeled gp120s with CD4 or CCR5 determined in the competition assays using either the anti-CD4 mAb Q4120 or ^35^S-gp120 #34 as tracer, respectively. ^**(a)**^ n.d.: Not determined because the low yields of ^35^S-gp120 #1 production did not allow the saturation binding experiments to be performed. ^**(b)**^ n.s.: Specific binding was not saturable over the range of the gp120 concentrations tested. ^**(c)**^ Shown are the IC_50_ values deduced from displacement of ^35^S-gp120 #34 binding by unlabelled gp120 #50 to high(H)- and low(L)- affinity CCR5. ^**(d)**^ Shown is the mean IC_50_ value deduced from the competition experiments of ^35^S-gp120 #34 binding by unlabelled gp120 #10. ^**(e)**^ The K_D_ values are deduced from the saturation binding experiments of ^35^S-gp120 #25 or #34 to membranes from HEK 293 cells expressing SNAP/FLAG (S/F)-tagged WT-CCR5 or L196K-CCR5. Results represent means ± SD of at least 3 independent experiments performed in duplicate.
(DOCX)

S1 Text Distinct HIV-1 gp120s differentially interact with antigenically distinct populations of CCR5. This text is related to S3A, S3B, S3C and S3D Fig.
(DOCX)

S2 Text Related to the competition experiments of ^35^S-gp120 #34 binding by unlabeled gp120s presented in Fig 2. (DOCX)

S1 Fig Binding of ^35^S-gp120s to intact HEK-CD4 cells. Experiments were carried out as in Fig 1F using 1 x 10^5^ cells in the assay buffer. A representative experiment out of two independent determinations is shown.
(PPTX)

S2 Fig The levels of gp120 binding to CCR5 vary differentially between different cell-types. **A** Specific binding of 10 nM of the indicated ^35^S-gp120s (+ 200 nM sCD4) to membranes from HEK-R5 cells or the CD4 negative, human primary glioblastoma cell line U87 in which we ectopically expressed CCR5 (U87-R5 cells). U87-R5 cells showing comparable labeling with the anti-CCR5 mAb 2D7 as compared to HEK-R5 cells were selected for these experiments. Results are expressed as fold-change of gp120 binding relative to specific binding of gp120 #1 to HEK-R5 membranes. Means ± SEM of four determinations with two distinct membrane preparations and two distinct lots of purified gp120s are shown. NSB, determined with 10 μM MVC, was consistently 1.2–1.7-fold lower on U87 than on HEK membranes. Panels **B** and **C** represent similar experiments as in **A** but using membranes from or intact CD4+ T-lymphocytes or MDMs. Fold-changes of gp120 #25 binding relative to gp120 #34 are shown. NSB weakly differed between intact cells and membranes and represented about 50% of total binding for both gp120s in the case of T-cells. With MDMs, this value approximated 50–60% and 70–80% for gp120 #34 and #25, respectively. These differences owed to lower specific binding of gp120 #25 *vs* gp120 #34, and not to differences in NSB between both gp120s. Results are means ± SEM of three independent experiments that were performed with the blood cells from three different healthy donors. The amounts of gp120 #34-binding receptors/cell from one individual to another ranged between 1935 and 2226 and between 2183 and 3579 on T-cells and MDMs, respectively. These cells thus express 10- to 20-fold lower amounts of CCR5 than HEK-R5 cells (compare with Fig 1E). The amounts of gp120 #34-binding receptors on membranes from T-cells and MDMs were 0.18–0.66 and 0.12–0.48 pmole/mg, respectively. * *P* < 0.05; ** *P* < 0.01; *** *P* < 0.001; ****P < 0.0001 compared to binding to HEK-R5 membranes (**A)** or to binding of ^35^S-gp120 #34 (**B**, **C**) in two-tailed Student *t* test.
(PPTX)

S3 Fig Different HIV-1 gp120s differentially recognize antigenically distinct populations of CCR5 in a cell-type dependent manner. **A** The anti-CCR5 mAbs CTC5, 2D7 and 45531 used in the displacement experiments of ^35^S-gp120 binding map distinct epitopes of CCR5. **B** Theoretical picture of gp120 binding competition by mAbs. In these experiments, assuming that mAbs and gp120s compete for binding to a single binding site, the law of mass action predicts that specific binding of gp120s diminishes from 90% to 10% with a two-log increase of the mAb concentration. **C** Binding of ^35^S-gp120s to HEK-R5 membranes was measured in the presence of the different mAbs used at two distinct concentrations (in μg/ml), one equal to their reported K_D_ for CCR5 [11] (hatched bars), the other being saturating (filled bars). Results (means ± SEM of 4 independent experiments performed in duplicate) were normalized for non-specific binding (0%) and specific binding in the absence of mAbs (100%, black bars). **D** Similar experiments as in **C** were performed using U87-R5 membranes. **E** Effects of saturating concentrations of anti-CCR5 mAbs CTC5, 2D7 and 45531 on infection of U87-CD4-CCR5 cells by equal amounts (100 ng Gag p24) of virus clones pseudotyped with different R5 Envs. Results represent means ± SEM of two independent experiments performed in duplicate, and are expressed as percent infection relative to control infection measured in the absence of mAbs (100%, black bars). Infectivities were determined by measuring the luciferase activity in the lysates of infected cells 48 h post-infection. Results also show that the viruses are equally sensitive to inhibition by 10 μM maraviroc (MVC), thus ruling out that they interact with MVC-low affinity conformations of CCR5.
(PPTX)

S4 Fig Saturation and competition binding experiments of gp120s from the Bx08 and 1f HIV-1 strains to membranes from HEK-R5 cells. **A** The saturation experiments showed that ^35^S-gp120 1f has a three-fold lower B_max_ value compared to ^35^S-gp120 Bx08. Despite this, however, unlabeled gp120 1f produced full displacement of ^35^S-gp120 Bx08 in competition experiments (**C**). The competition curve has a hill slope value n_H_ of 1.03 ± 0.1 (*vs* n_H_ = 1.05 ± 0.09 in the case of the homologous competition between ^35^S-labeled and unlabeled gp120 Bx08). This suggests that both gp120s bind to a single class of receptors. Moreover, from the competition curves, we deduced K_i_ values for the unlabeled proteins (K_i_ = 9.05 ± 0.7 and 5.85 ± 0.7 for gp120 Bx08 and 1f, respectively) that are similar to the K_D_ values determined in the saturation assays (panel B), suggesting that competitive inhibition takes place between ^35^S-gp120 Bx08 and unlabeled gp120 1f or Bx08. Considered altogether, these data strongly suggests that while both gp120s display divergent binding levels in the saturation binding experiments **(A)**, they recognize the same receptors. Results are means ± SEM of two independent experiments.
(PPTX)

S5 Fig Fusion kinetics of virus #25 or #34 with WT-CCR5- or L196K-CCR5-expressing A3.01 T-cells. Each of the fusion kinetics shown in Fig 5 was expressed as percent of fusion relative to the maximal extent of fusion measured at t = 360 min. Results are presented for all cells (**A**) or the cells expressing high (**B**), intermediate (**C**) or low (**D**) amounts of receptors at the cell surface. They show that while the fusion efficacies (*i*.*e*. F_max_) can differ between virus #25 and #34, depending on the nature of the receptor as well as the receptor expression level (Fig 5), fusion of both viruses with the target cells proceed at the same speed.
(PPTX)

S6 Fig Effects of WT- or L196K-CCR5 on entry of BlaM-vpr containing virus #25 or #34 into HEK-CD4 cells. CD4-expressing HEK 293 T cells (described in ref 42) in 6-well plates (5 x 10^5^ cells / well) were incubated for 48 h with 25 or 400 ng Gag p24 of FLAG/SNAP-tagged WT-CCR5- or L196K-CCR5-expressing lentiviral particles, respectively. (**A**) Expression levels of receptors on transduced cells were then verified by incubating cells for 30 min at 4°C in FACS buffer containing unconjugated anti-Flag mAb M2 (2 μg/ml, left panel) or anti-CCR5 mAb 2D7 (2.5 μg/ml, right panel). Cells were then washed twice and then further incubated in FACS buffer containing AlexaFluor 647-conjugated goat anti-mouse IgG (GAM) (Life Technologies). Data were acquired out on a FACSCanto flow cytometer and analyzed using FlowJo. Magenta and blue histograms represent fluorescence signals for L196K-CCR5 and WT-CCR5, respectively. Background signal of untransduced cells labeled with GAM alone is represented as red histograms. (**B**) In parallel, transduced cells (1.5 x 10^5^) were incubated for 3h in the presence of 50 ng Gag 24 of BlaM-vpr-containing virus #25 or #34. Cells were then further incubated for 2 h with the CCF2/AM dye. Enzymatic cleavage of CCF2 by β-lactamase in the target cells was analyzed by flow cytometry (FACSCanto, BD Biosciences). Results (means ± SEM, n = 3) are expressed as percent fusion, *i*.*e*. percent of cells expressing cleaved CCF2. * *P*<0.05; ** *P* <0.01, in unpaired two-tailed Student *t* test.
(PPTX)

S7 Fig Amino acid sequence alignment of the gp120s used in this study. The HIV-1 gp120 consists of constant regions separated by five variable domains (V1 to V5). The V3 loop (red box) and regions forming the bridging sheet (green-colored) that play a crucial role in binding to CCR5 are shown. Numbering of amino acids is performed relative to the HxB2 reference sequence.
(PPTX)
