# Supplementary material for: CCR5 structural plasticity shapes HIV-1 phenotypic properties
Source: PLoS Pathog. 2018 Dec 6;14(12):e1007432. doi: 10.1371/journal.ppat.1007432 (PMC6283471; doi:10.1371/journal.ppat.1007432)
Supplement: S1 Table — The KD values represent the equilibrium dissociation constants of the 35S-gp120-sCD4/CCR5 complexes deduced in the saturation binding experiments to membranes from HEK 293T cells expressing CCR5 (HEK-R5 cells). The Bmax values represent the maximum numbers of receptors binding the 35S-gp120-sCD4 complexes in these experiments. KIQ4120 and KIgp120 #34 represent the equilibrium dissociation constants for interaction of the indicated unlabeled gp120s with CD4 or CCR5 determined in the competition assays using either the anti-CD4 mAb Q4120 or 35S-gp120 #34 as tracer, respectively. (a) n.d.: Not determined because the low yields of 35S-gp120 #1 production did not allow the saturation binding experiments to be performed. (b) n.s.: Specific binding was not saturable over the range of the gp120 concentrations tested. (c) Shown are the IC50 values deduced from displacement of 35S-gp120 #34 binding by unlabelled gp120 #50 to high(H)- and low(L)- affinity CCR5. (d) Shown is the mean IC50 value deduced from the competition experiments of 35S-gp120 #34 binding by unlabelled gp120 #10. (e) The KD values are deduced from the saturation binding experiments of 35S-gp120 #25 or #34 to membranes from HEK 293 cells expressing SNAP/FLAG (S/F)-tagged WT-CCR5 or L196K-CCR5. Results represent means ± SD of at least 3 independent experiments performed in duplicate. (DOCX) [file ppat.1007432.s001.docx]

**S1 Table. Binding parameters of the different HIV-1 gp120s used in the study.**

| **gp120 #** | **K_D_ (nM)** | **B_max_ (pmole/mg)** | **K_I_^Q4120^ (nM)** | **K_I_^gp120 #34^ (nM)** | **K_D_ (nM)**  **(S/F-WT)^(e)^** | **K_D_ (nM)**  **(S/F-196K)^(e)^** |
| --- | --- | --- | --- | --- | --- | --- |
| **1** | n.d.**^(a)^** | n.d.**^(a)^** | 7.8 ± 6.2 | - | - | - |
| **10** | n.s.**^(b)^** | n.s.**^(b)^** | 7.3 ± 4.1 | 37 ± 10**^(d)^** | - | - |
| **25** | 7.4 ± 2.4 | 0.6 ± 0.1 | 7.3 ± 3.1 | 3.6 ± 1.4 | 13.6 ± 3 | 12.5 ± 1.9 |
| **34** | 6.9 ± 1.1 | 1.3 ± 0.2 | 10.5 ± 6.1 | 4.6 ± 0.3 | 14.2 ± 5.9 | 10.8. ± 3.3 |
| **38** | 17.4 ± 1.7 | 0.7 ± 0.03 | - | - | - | - |
| **48** | 10 ± 1.1 | 0.7 ± 0.1 | - | - | - | - |
| **50** | 9.7 ± 2.2 | 0.5 ± 0.1 | 13.9 ± 4.6 | (H) 0.5 ± 0.3**^(c)^**  (L) 81 ± 35**^(c)^** | - | - |
| **58** | n.s.**^(b)^** | n.s.**^(b)^** | 12.6 ± 7.1 | > 100 | - | - |
| **59** | 33.2 ± 17.4 | 0.3 ± 0.1 | - | - | - | - |
| **Bx08** | 14.1 ± 2.5 | 1.2 ± 0.2 | - | - | - | - |

The K_D_ values represent the equilibrium dissociation constants of the ^35^S-gp120-sCD4/CCR5 complexes deduced in the saturation binding experiments to membranes from HEK 293T cells expressing CCR5 (HEK-R5 cells). The B_max_ values represent the maximum numbers of receptors binding the ^35^S-gp120-sCD4 complexes in these experiments.

K_I_^Q4120^ and K_I_^gp120 #34^ represent the equilibrium dissociation constants for interaction of the indicated unlabeled gp120s with CD4 or CCR5 determined in the competition assays using either the anti-CD4 mAb Q4120 or ^35^S-gp120 #34 as tracer, respectively.

**^(a)^** n.d.: Not determined because the low yields of ^35^S-gp120 #1 production did not allow the saturation binding experiments to be performed.

**^(b)^** n.s.: Specific binding was not saturable over the range of the gp120 concentrations tested.

**^(c)^** Shown are the IC_50_ values deduced from displacement of ^35^S-gp120 #34 binding by unlabelled gp120 #50 to high(H)- and low(L)- affinity CCR5.

**^(d)^** Shown is the mean IC_50_ value deduced from the competition experiments of ^35^S-gp120 #34 binding by unlabelled gp120 #10.

**^(e)^** The K_D_ values are deduced from the saturation binding experiments of ^35^S-gp120 #25 or #34 to membranes from HEK 293 cells expressing SNAP/FLAG (S/F)-tagged WT-CCR5 or L196K-CCR5.

Results represent means ± SD of at least 3 independent experiments performed in duplicate.
