# Supplementary material for: CCR5 structural plasticity shapes HIV-1 phenotypic properties
Source: PLoS Pathog. 2018 Dec 6;14(12):e1007432. doi: 10.1371/journal.ppat.1007432 (PMC6283471; doi:10.1371/journal.ppat.1007432)
Supplement: S1 Text — This text is related to S3A, S3B, S3C and S3D Fig. (DOCX) [file ppat.1007432.s002.docx]

**S1 Text.** **Distinct HIV-1 gp120s differentially interact with antigenically distinct populations of CCR5 (related to S3A, S3B, S3C and S3D Fig)**

To investigate whether the different gp120s bind differentially to distinct antigenic conformations of CCR5, we performed competition binding experiments of the ^35^S-gp120s to HEK-R5 or U87-R5 cells by the anti-CCR5 mAbs CTC5, 2D7 or 45531. These mAbs react with distinct regions of CCR5 (**S3A Fig**) and do not target the same CCR5 conformations [1, 2]. We used these mAbs at two distinct concentrations, one equal to their reported K_D_ for CCR5 [1], the other being saturating. Three main conclusions emerged from these experiments (**S3C** and **S3D Fig**). First, different gp120s may be differentially sensitive to inhibition by some of the mAbs. For instance, 1.5 nM 2D7 half-inhibited the binding of ^35^S-gp120 #50 to HEK-R5 cells but poorly affected the binding of ^35^S-gp120 #10 and almost completely abolished that of gp120 #38 (**S3C Fig**). Assuming that inhibition of gp120 binding by 2D7 obeys the law of mass action, this implies that the IC_50_ values for half-maximal inhibition of gp120 binding by 2D7 (*i.e.* the inhibition potencies of 2D7) vary over two orders of magnitude depending upon the nature of the gp120 (**S3B Fig**). In other words, this strongly suggests that gp120s #50, #38 and #10 bind differentially distinct CCR5 populations, which differ in their affinity for 2D7. To a lesser extent, differential sensitivities of the different gp120s to inhibition by the other two mAbs could also be observed (*e.g.* in **S3C Fig**, gp120s #58, 50 and 25 are the most resistant proteins to mAb 45531, and gp120s #10, 25 and 50 resist better to mAb CTC5 than other gp120s). As a consequence, the differences in inhibition efficiencies between the 3 mAbs may vary from one gp120 to another.

Second, we found that sensitivity of the gp120s to the mAbs varies between HEK-R5 (**S3C Fig**) and U87-R5 (**S3D Fig)** cells. Most of the gp120s was less sensitive to 2D7 in U87-R5 cells, while the reverse was generally observed with CTC5 and 45531, suggesting that the gp120s do not bind the same CCR5 conformations in both cell types. Interestingly, the mAbs showed differences in their inhibitory capacity from one gp120 to another that fluctuated less in U87-R5 than in HEK-R5 cells. This is particularly true for 2D7, which inhibited similarly most of the tested gp120s in U87-R5 cells. Actually, some of the gp120s (*e.g.* gp120 #25, #34 and #58) showed similar sensitivity to the mAbs in U87-R5 cells, while they displayed great differences in their binding capacity to these cells (**S2 Fig**). This suggests that distinct CCR5 forms can bind differentially distinct gp120s while having similar antigenicity. Third, binding of some of the gp120s was only partially inhibited by the mAbs (*e.g.* gp120 #10 with CTC5 in HEK-R5 cells, gp120s #38 and 48 with CTC5 in U87-R5 cells), suggesting that these gp120s recognize at least two distinct CCR5 populations, one interacting with the mAb, the other that does not. Alternatively, the remaining binding of a gp120 in the presence of a saturating concentration of a mAb could reflect that the gp120 can maintain interaction with the mAb-bound form of the receptor, but with lower affinity as compared to interaction with the free receptor. This could be accomplished considering that the gp120 interacts with at least two distinct regions on CCR5, one overlapping with the mAb binding site, the other that does not.

These results considered altogether indicate that the different gp120s may target differentially distinct antigenic populations of CCR5, and it is a possibility that this contributes to some extent to their divergent binding levels to CCR5-expressing cells (**Fig 1** and **S2 Fig**). However, the differences in the CCR5 binding properties between the distinct gp120s cannot solely be explained by differential recognition of distinct antigenic forms of CCR5. Indeed, distinct CCR5 populations can vary in the nature of the gp120s to which they bind while sharing similar antigenic properties.

**References**

1. Berro R, Klasse PJ, Lascano D, Flegler A, Nagashima KA, Sanders RW, et al. Multiple CCR5 conformations on the cell surface are used differentially by human immunodeficiency viruses resistant or sensitive to CCR5 inhibitors. Journal of virology. 2011;85(16):8227-40. PubMed PMID: 21680525.

2. Fox JM, Kasprowicz R, Hartley O, Signoret N. CCR5 susceptibility to ligand-mediated down-modulation differs between human T lymphocytes and myeloid cells. Journal of leukocyte biology. 2015;98(1):59-71. Epub 2015/05/10. doi: 10.1189/jlb.2A0414-193RR. PubMed PMID: 25957306.
