# Supplementary material for: CCR5 structural plasticity shapes HIV-1 phenotypic properties
Source: PLoS Pathog. 2018 Dec 6;14(12):e1007432. doi: 10.1371/journal.ppat.1007432 (PMC6283471; doi:10.1371/journal.ppat.1007432)
Supplement: S2 Text — (DOCX) [file ppat.1007432.s003.docx]

**S2 Text.** **Related to the competition experiments of ^35^S-gp120 #34 binding by unlabeled gp120s presented in Fig 2.**

To evidence more directly that different gp120s bind to/stabilize distinct CCR5 subsets, we measured the binding of ^35^S-gp120 #34 to HEK-R5 membranes in the presence of unlabeled gp120 #10, #25, #34, #50 or #58 (**Fig 2A**, **2B and 2C**). From the homologous competition between ^35^S-gp120 #34 and unlabeled gp120 #34 (**Fig 2A**), we calculated a K_I_ value for the unlabeled protein that is comparable to the K_D_ value determined for ^35^S-gp120 #34 in the saturation binding assay (**S1 Table**). The Hill slope n_H_, describing the steepness of the competition curve, approximated unity (n_H_ = -0.93±0.06), indicating that ^35^S-gp120 #34 and its unlabeled counterpart compete for binding to a unique class of receptors, as expected. By contrast, shallower competition curves were obtained using unlabeled gp120 #50 or #10 as competitors (n_H_ = -0.5 and -0.57, respectively) (**Fig 2B** and **2C**), suggesting that each of these two gp120s recognizes multiple ^35^S-gp120 #34-binding receptors with different affinities. Actually, a two-site competitive binding model best described the competition curve between ^35^S-gp120 #34 and gp120 #50 (legend of **Fig 2B**), indicating the presence of two distinct ^35^S-gp120 #34-binding receptor populations, one with nanomolar affinity for gp120 #50, the other with substantially lower affinity (**S1 Table**). Gp120 #58 failed to displace the binding of ^35^S-gp120 #34, indicating that gp120 #58 has low affinity for the ^35^S-gp120 #34-binding receptors. Taken together, these results further emphasize that some of the different gp120s primarily differ in that they do not recognize the same CCR5 populations.

Gp120 #25 and gp120 #34 were equally potent and efficient in displacing the binding of ^35^S-gp120 #34 (**Fig 2A**). This result was unanticipated because gp120 #25 binds less receptors than gp120 #34 in the saturation experiments (**Fig 1A** and **1B**). Similar observations with other gp120s are presented in **S4 Fig**. Similarly to gp120 #25, other GPCR ligands fully displace the binding of radioactive tracers while labeling lower amounts of receptors in saturation binding assays [1]. In that case, results were interpreted in terms of those ligands transmitting negative cooperative effects between units of receptor oligomers [1, 2]. Our results, however, do not support the possibility that gp120 #25 inhibits the binding of ^35^S-gp120 #34 by such an allosteric mechanism. Indeed, the deduced K_I_ and K_D_ values for gp120 #25 are similar (**S1 Table**), indicating that competitive inhibition likely takes place [1]. In addition, gp120 #25 displaces ^35^S-gp120 #34 binding with a n_H_ close to unity (n_H_ = -0.91±0.1), indicating that both ligands compete for binding to a single class of receptors. In the context where CCR5 exists as oligomers [2, 3], a model recapitulating the above results considers that gp120s establish different interaction stoichiometries with the CCR5 oligomers. In the simplest version of this model (see **Fig 4**, left panel), gp120 could form both 1:2 and 2:2 stoichiometric complexes with the CCR5 dimers, the frequency of each of these two situations depending on the nature of the gp120. In the first situation, a single gp120 would interact concomitantly with both protomers of the CCR5 dimer, in line with gp120s being multivalent ligands that interact concomitantly with distant regions of CCR5 [4-6]. Each protomer would provide different interaction sites for the gp120 (*e.g.* the N-tail could originate from protomer 1 and ECL2 from protomer 2), hence forming the whole binding site for the glycoprotein. In the second situation, the two protomers would provide two identical and independent binding sites per dimer for the gp120. We propose that gp120 #25 favors the 1:2 stoichiometry at the expense of the 2:2 stoichiometry, and conversely for gp120 #34. This would explain how gp120 #25 could inhibit the binding of gp120 #34 by a competitive mechanism while showing a lower B_max_ value in saturation binding assays. This model also considers oligomeric CCR5 as a particular CCR5 population that could play a major role in diversification of the modes of gp120/CCR5 interactions.

**References**

1. Armstrong D, Strange PG. Dopamine D2 receptor dimer formation: evidence from ligand binding. The Journal of biological chemistry. 2001;276(25):22621-9. Epub 2001/03/30. doi: 10.1074/jbc.M006936200. PubMed PMID: 11278324.

2. Springael JY, Le Minh PN, Urizar E, Costagliola S, Vassart G, Parmentier M. Allosteric modulation of binding properties between units of chemokine receptor homo- and hetero-oligomers. Molecular pharmacology. 2006;69(5):1652-61. PubMed PMID: 16467191.

3. Issafras H, Angers S, Bulenger S, Blanpain C, Parmentier M, Labbe-Jullie C, et al. Constitutive agonist-independent CCR5 oligomerization and antibody-mediated clustering occurring at physiological levels of receptors. The Journal of biological chemistry. 2002;277(38):34666-73.

4. Cormier EG, Dragic T. The crown and stem of the V3 loop play distinct roles in human immunodeficiency virus type 1 envelope glycoprotein interactions with the CCR5 coreceptor. Journal of virology. 2002;76(17):8953-7. PubMed PMID: 12163614.

5. Huang CC, Lam SN, Acharya P, Tang M, Xiang SH, Hussan SS, et al. Structures of the CCR5 N terminus and of a tyrosine-sulfated antibody with HIV-1 gp120 and CD4. Science. 2007;317(5846):1930-4. PubMed PMID: 17901336.

6. Huang CC, Tang M, Zhang MY, Majeed S, Montabana E, Stanfield RL, et al. Structure of a V3-containing HIV-1 gp120 core. Science. 2005;310(5750):1025-8. PubMed PMID: 16284180.
