# Supplementary figures and images for: CCR5 structural plasticity shapes HIV-1 phenotypic properties
Source: PLoS Pathog. 2018 Dec 6;14(12):e1007432. doi: 10.1371/journal.ppat.1007432 (PMC6283471; doi:10.1371/journal.ppat.1007432)

## Slide 1
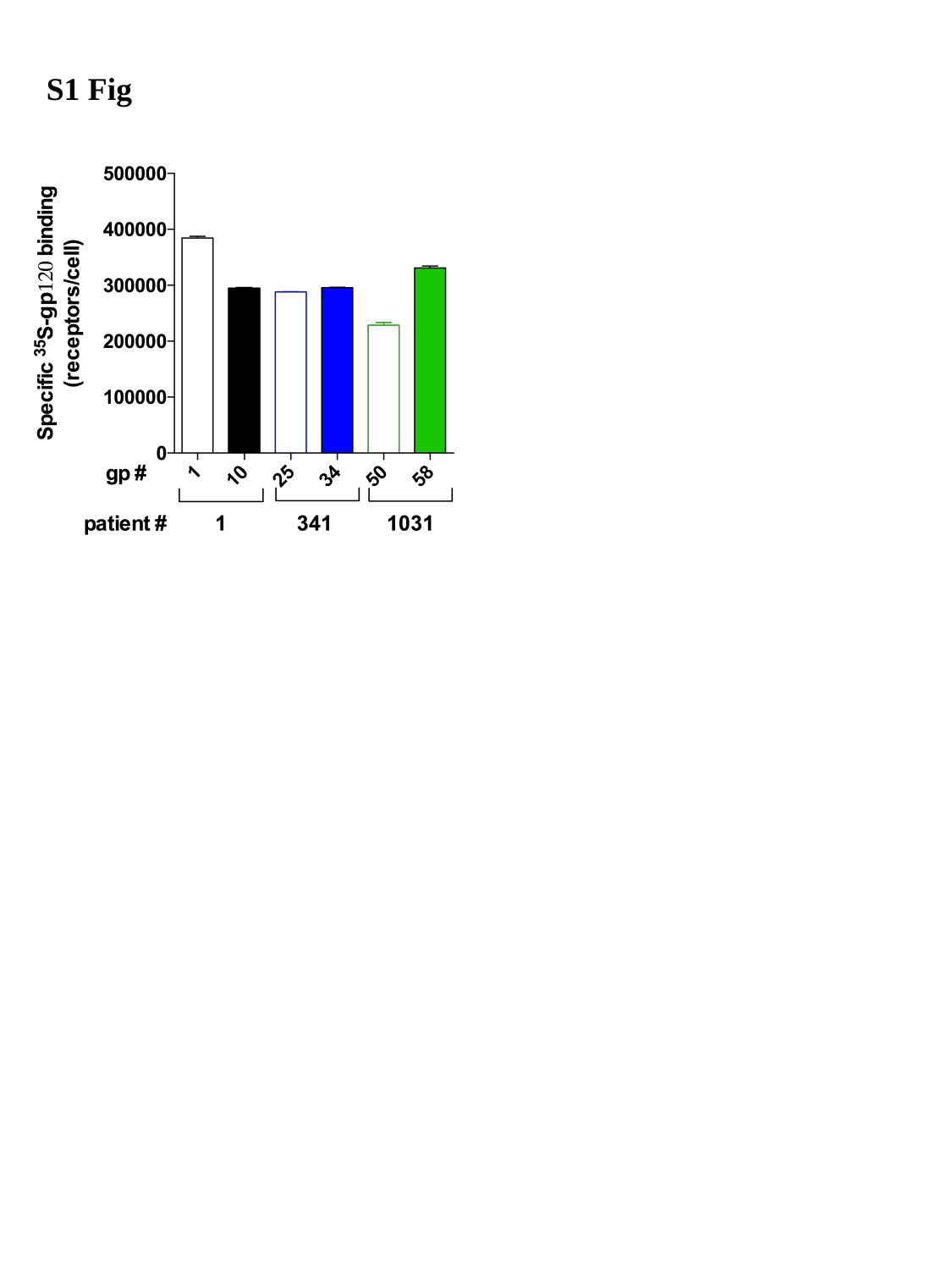

S1 Fig

Supplement: S1 Fig — Experiments were carried out as in Fig 1F using 1 x 10^5^ cells in the assay buffer. A representative experiment out of two independent determinations is shown. (PPTX) [file ppat.1007432.s004.pptx]

## Slide 1
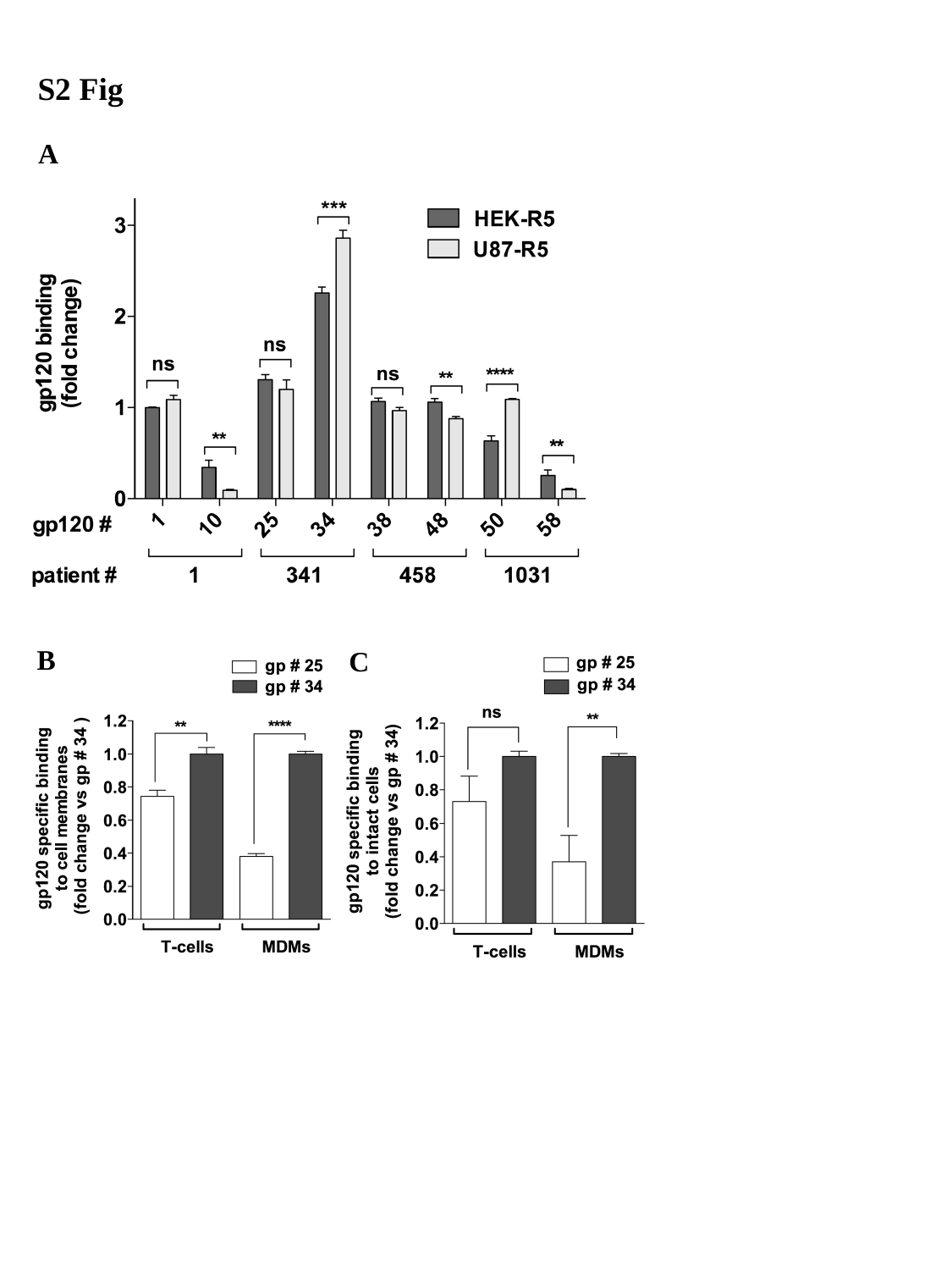

S2 Fig
A
B
C

Supplement: S2 Fig — A Specific binding of 10 nM of the indicated 35S-gp120s (+ 200 nM sCD4) to membranes from HEK-R5 cells or the CD4 negative, human primary glioblastoma cell line U87 in which we ectopically expressed CCR5 (U87-R5 cells). U87-R5 cells showing comparable labeling with the anti-CCR5 mAb 2D7 as compared to HEK-R5 cells were selected for these experiments. Results are expressed as fold-change of gp120 binding relative to specific binding of gp120 #1 to HEK-R5 membranes. Means ± SEM of four determinations with two distinct membrane preparations and two distinct lots of purified gp120s are shown. NSB, determined with 10 μM MVC, was consistently 1.2–1.7-fold lower on U87 than on HEK membranes. Panels B and C represent similar experiments as in A but using membranes from or intact CD4+ T-lymphocytes or MDMs. Fold-changes of gp120 #25 binding relative to gp120 #34 are shown. NSB weakly differed between intact cells and membranes and represented about 50% of total binding for both gp120s in the case of T-cells. With MDMs, this value approximated 50–60% and 70–80% for gp120 #34 and #25, respectively. These differences owed to lower specific binding of gp120 #25 vs gp120 #34, and not to differences in NSB between both gp120s. Results are means ± SEM of three independent experiments that were performed with the blood cells from three different healthy donors. The amounts of gp120 #34-binding receptors/cell from one individual to another ranged between 1935 and 2226 and between 2183 and 3579 on T-cells and MDMs, respectively. These cells thus express 10- to 20-fold lower amounts of CCR5 than HEK-R5 cells (compare with Fig 1E). The amounts of gp120 #34-binding receptors on membranes from T-cells and MDMs were 0.18–0.66 and 0.12–0.48 pmole/mg, respectively. * P < 0.05; ** P < 0.01; *** P < 0.001; ****P < 0.0001 compared to binding to HEK-R5 membranes (A) or to binding of 35S-gp120 #34 (B, C) in two-tailed Student t test. (PPTX) [file ppat.1007432.s005.pptx]

## Slide 1
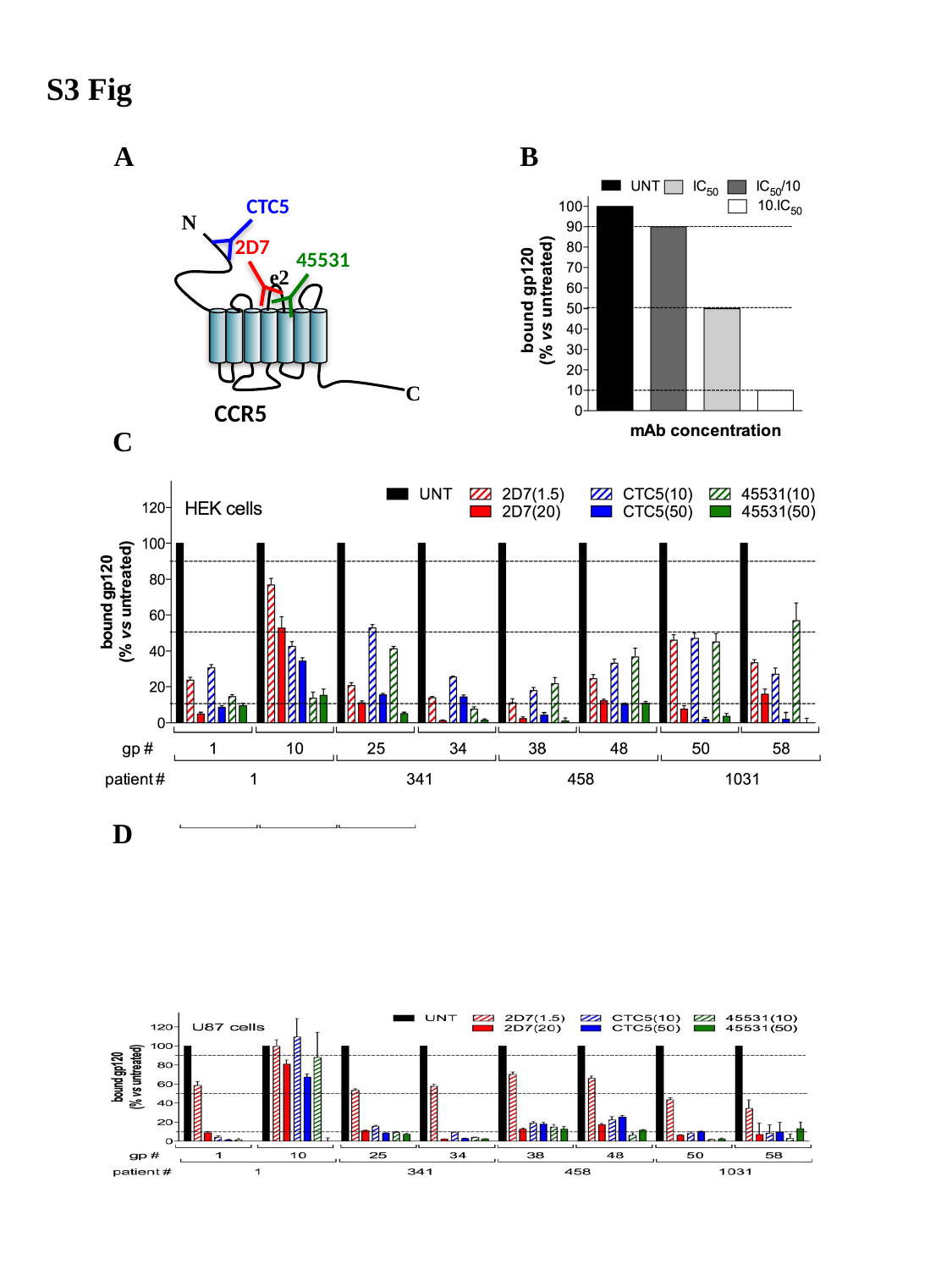

S3 Fig
A
B
CTC5
N
2D7
45531
e2
C
CCR5
C
D

## Slide 2
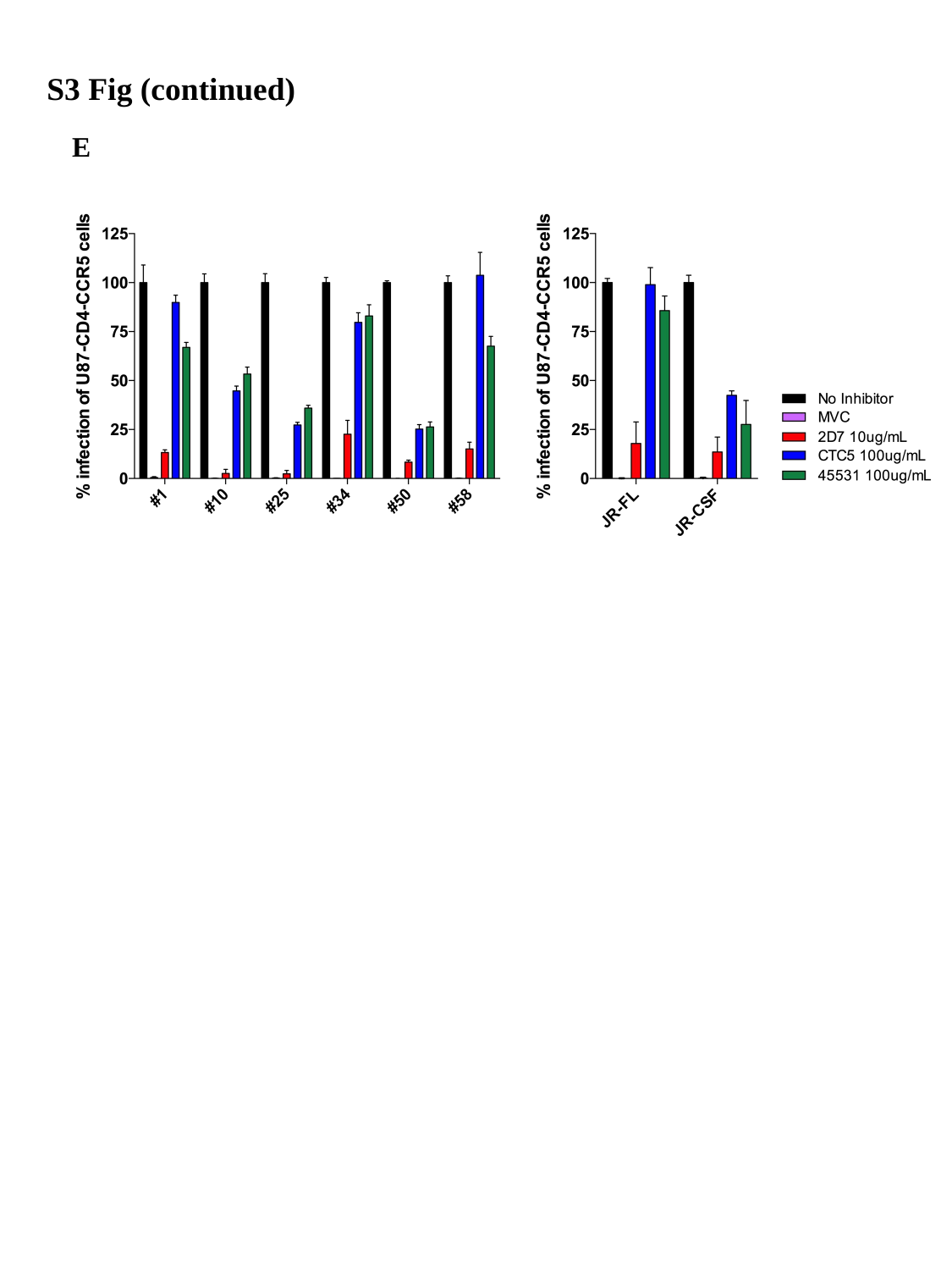

S3 Fig (continued)
E

Supplement: S3 Fig — A The anti-CCR5 mAbs CTC5, 2D7 and 45531 used in the displacement experiments of 35S-gp120 binding map distinct epitopes of CCR5. B Theoretical picture of gp120 binding competition by mAbs. In these experiments, assuming that mAbs and gp120s compete for binding to a single binding site, the law of mass action predicts that specific binding of gp120s diminishes from 90% to 10% with a two-log increase of the mAb concentration. C Binding of 35S-gp120s to HEK-R5 membranes was measured in the presence of the different mAbs used at two distinct concentrations (in μg/ml), one equal to their reported KD for CCR5 [11] (hatched bars), the other being saturating (filled bars). Results (means ± SEM of 4 independent experiments performed in duplicate) were normalized for non-specific binding (0%) and specific binding in the absence of mAbs (100%, black bars). D Similar experiments as in C were performed using U87-R5 membranes. E Effects of saturating concentrations of anti-CCR5 mAbs CTC5, 2D7 and 45531 on infection of U87-CD4-CCR5 cells by equal amounts (100 ng Gag p24) of virus clones pseudotyped with different R5 Envs. Results represent means ± SEM of two independent experiments performed in duplicate, and are expressed as percent infection relative to control infection measured in the absence of mAbs (100%, black bars). Infectivities were determined by measuring the luciferase activity in the lysates of infected cells 48 h post-infection. Results also show that the viruses are equally sensitive to inhibition by 10 μM maraviroc (MVC), thus ruling out that they interact with MVC-low affinity conformations of CCR5. (PPTX) [file ppat.1007432.s006.pptx]

## Slide 1
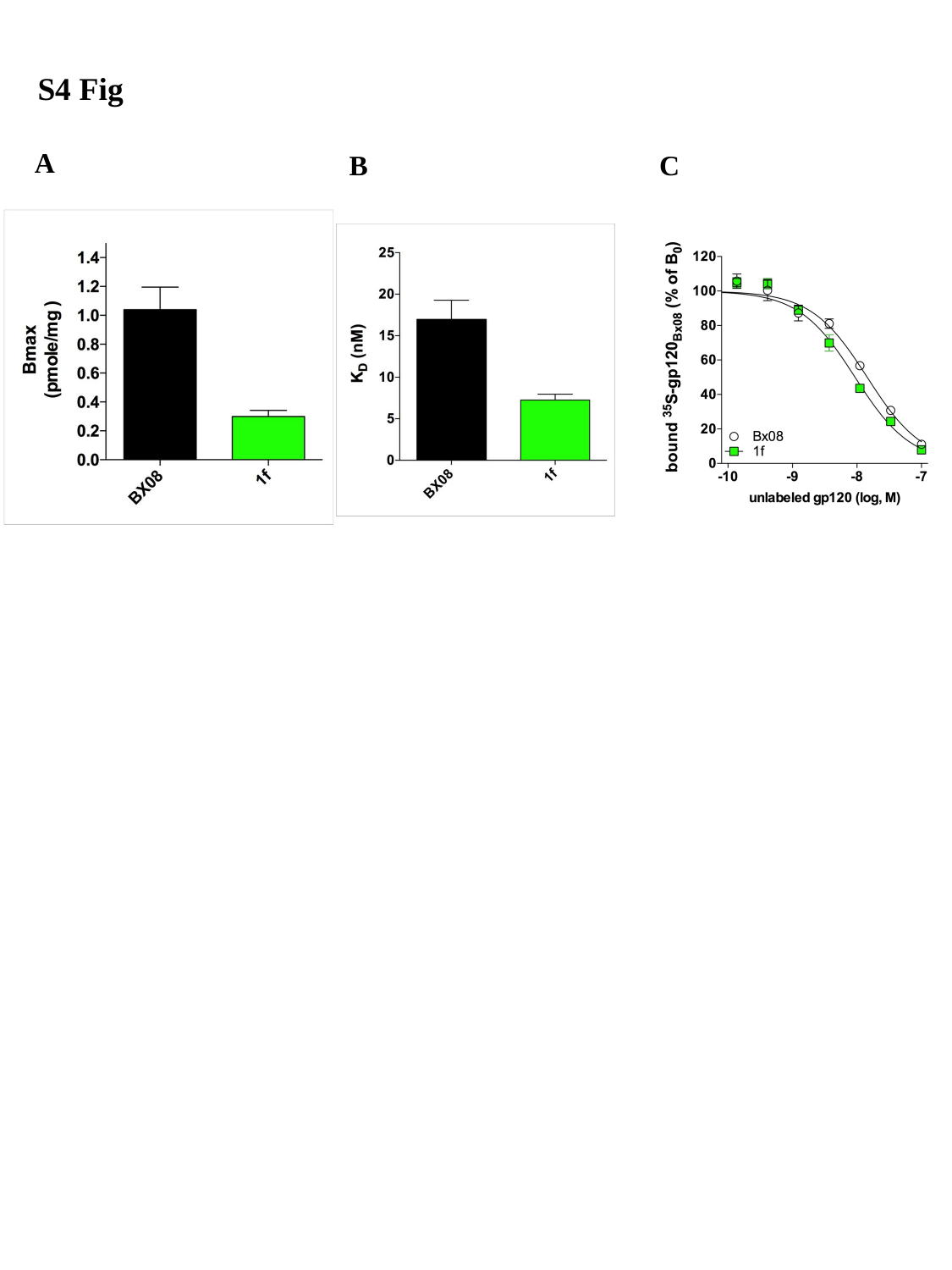

S4 Fig
A
B
C

Supplement: S4 Fig — A The saturation experiments showed that 35S-gp120 1f has a three-fold lower Bmax value compared to 35S-gp120 Bx08. Despite this, however, unlabeled gp120 1f produced full displacement of 35S-gp120 Bx08 in competition experiments (C). The competition curve has a hill slope value nH of 1.03 ± 0.1 (vs nH = 1.05 ± 0.09 in the case of the homologous competition between 35S-labeled and unlabeled gp120 Bx08). This suggests that both gp120s bind to a single class of receptors. Moreover, from the competition curves, we deduced Ki values for the unlabeled proteins (Ki = 9.05 ± 0.7 and 5.85 ± 0.7 for gp120 Bx08 and 1f, respectively) that are similar to the KD values determined in the saturation assays (panel B), suggesting that competitive inhibition takes place between 35S-gp120 Bx08 and unlabeled gp120 1f or Bx08. Considered altogether, these data strongly suggests that while both gp120s display divergent binding levels in the saturation binding experiments (A), they recognize the same receptors. Results are means ± SEM of two independent experiments. (PPTX) [file ppat.1007432.s007.pptx]

## Slide 1
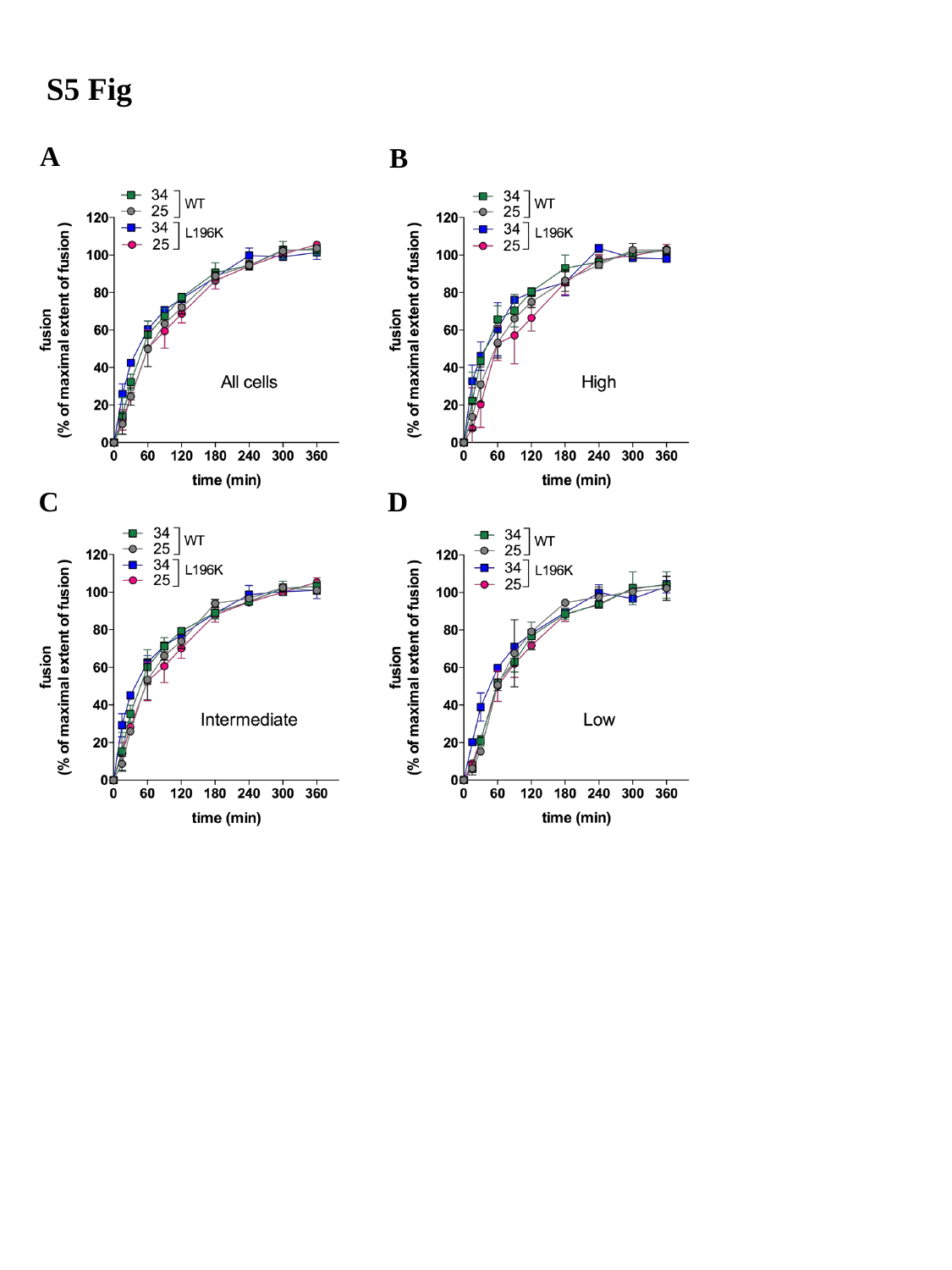

S5 Fig
A
B
C
D

Supplement: S5 Fig — Each of the fusion kinetics shown in Fig 5 was expressed as percent of fusion relative to the maximal extent of fusion measured at t = 360 min. Results are presented for all cells (A) or the cells expressing high (B), intermediate (C) or low (D) amounts of receptors at the cell surface. They show that while the fusion efficacies (i.e. Fmax) can differ between virus #25 and #34, depending on the nature of the receptor as well as the receptor expression level (Fig 5), fusion of both viruses with the target cells proceed at the same speed. (PPTX) [file ppat.1007432.s008.pptx]

## Slide 1
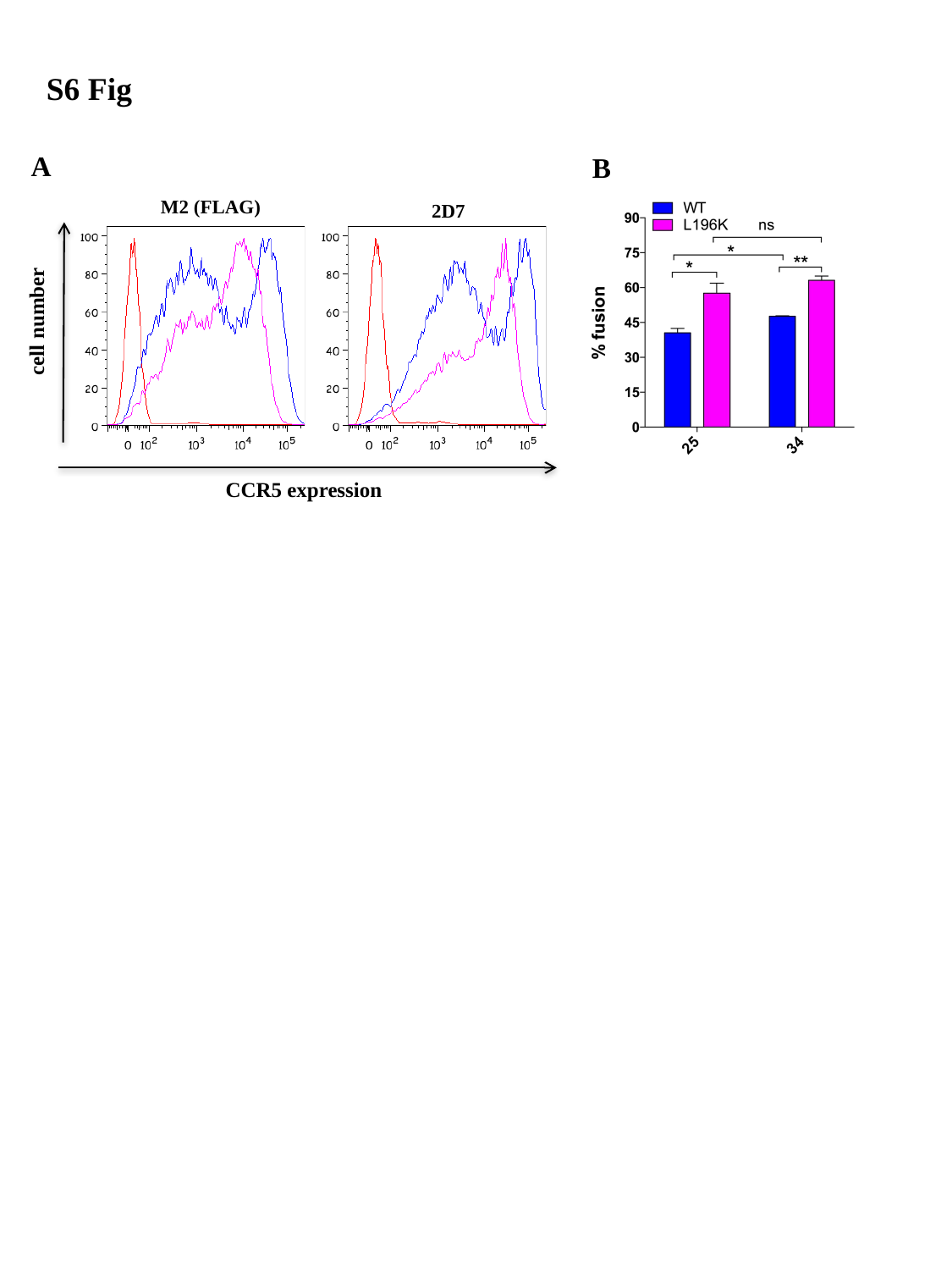

S6 Fig
A
B
M2 (FLAG)
2D7
cell number
CCR5 expression

Supplement: S6 Fig — CD4-expressing HEK 293 T cells (described in ref 42) in 6-well plates (5 x 10^5^ cells / well) were incubated for 48 h with 25 or 400 ng Gag p24 of FLAG/SNAP-tagged WT-CCR5- or L196K-CCR5-expressing lentiviral particles, respectively. (A) Expression levels of receptors on transduced cells were then verified by incubating cells for 30 min at 4°C in FACS buffer containing unconjugated anti-Flag mAb M2 (2 μg/ml, left panel) or anti-CCR5 mAb 2D7 (2.5 μg/ml, right panel). Cells were then washed twice and then further incubated in FACS buffer containing AlexaFluor 647-conjugated goat anti-mouse IgG (GAM) (Life Technologies). Data were acquired out on a FACSCanto flow cytometer and analyzed using FlowJo. Magenta and blue histograms represent fluorescence signals for L196K-CCR5 and WT-CCR5, respectively. Background signal of untransduced cells labeled with GAM alone is represented as red histograms. (B) In parallel, transduced cells (1.5 x 10^5^) were incubated for 3h in the presence of 50 ng Gag 24 of BlaM-vpr-containing virus #25 or #34. Cells were then further incubated for 2 h with the CCF2/AM dye. Enzymatic cleavage of CCF2 by β-lactamase in the target cells was analyzed by flow cytometry (FACSCanto, BD Biosciences). Results (means ± SEM, n = 3) are expressed as percent fusion, i.e. percent of cells expressing cleaved CCF2. * P<0.05; ** P <0.01, in unpaired two-tailed Student t test. (PPTX) [file ppat.1007432.s009.pptx]

## Slide 1
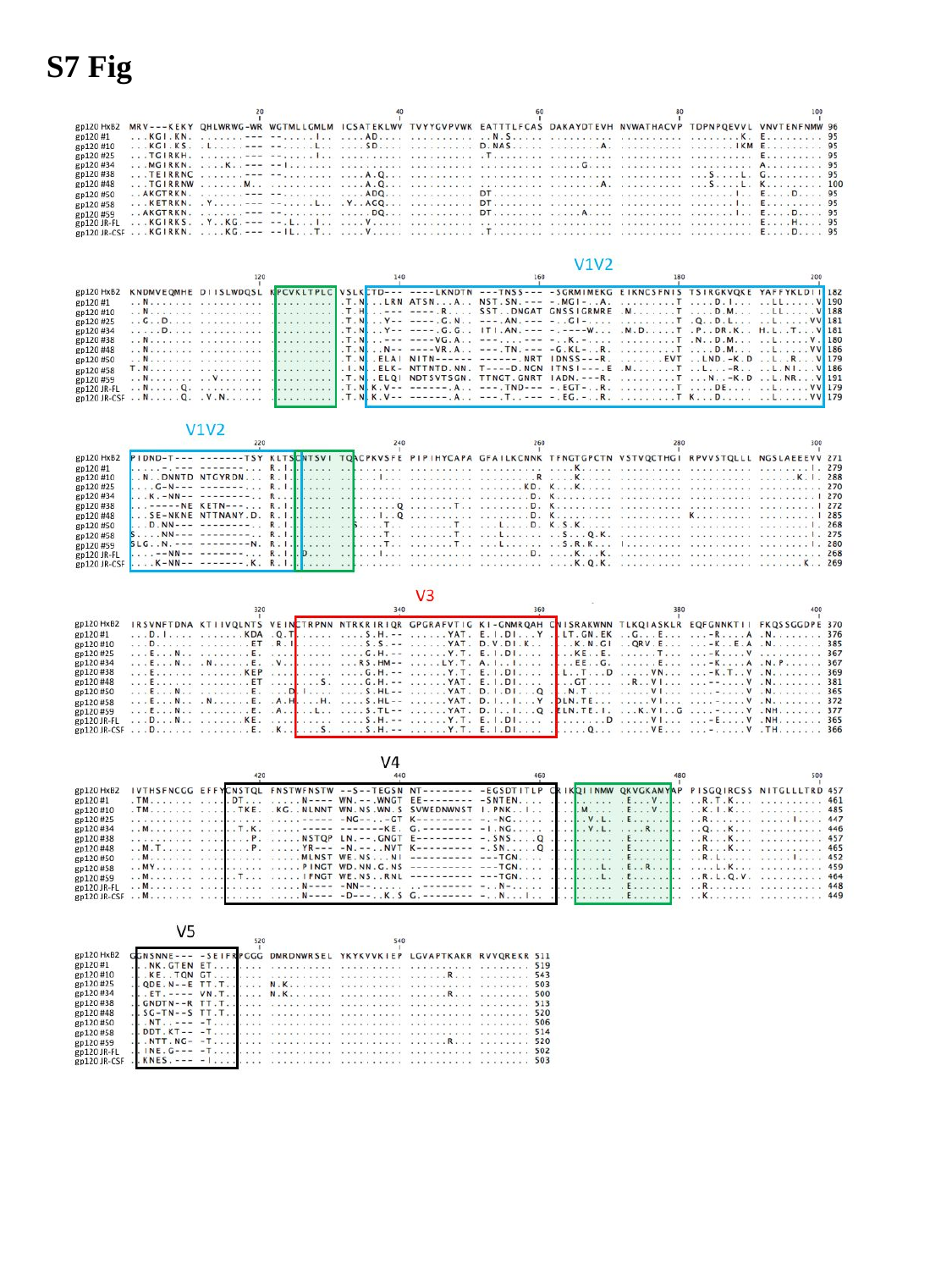

S7 Fig

Supplement: S7 Fig — The HIV-1 gp120 consists of constant regions separated by five variable domains (V1 to V5). The V3 loop (red box) and regions forming the bridging sheet (green-colored) that play a crucial role in binding to CCR5 are shown. Numbering of amino acids is performed relative to the HxB2 reference sequence. (PPTX) [file ppat.1007432.s010.pptx]
